# A new mayfly subfamily sheds light on the early evolution and Pangean origin of Baetiscidae (Insecta: Ephemeroptera)

**DOI:** 10.1038/s41598-024-51176-7

**Published:** 2024-01-18

**Authors:** Roman J. Godunko, Pavel Sroka

**Affiliations:** 1 Biology Centre of the Czech Academy of Sciences, Institute of Entomology, Branišovská 31, 37005 České Budějovice, Czech Republic; 2 Department of Invertebrate Zoology and Hydrobiology, Faculty of Biology and Environmental Protection, University of Lodz, Banacha 12/16, 90237 Łódź, Poland; 3 State Museum of Natural History, National Academy of Sciences of Ukraine, Teatralna 18, 79008 Lviv, Ukraine

**Keywords:** Entomology, Taxonomy, Palaeontology, Phylogenetics

## Abstract

The family Baetiscidae Edmunds & Traver, 1954 is recognisable among mayflies due to its bizarre larvae, equipped with a robust and spiked thoracic notal shield covering part of the abdomen up to sixth segment. Originally being described as extant species from the USA and Canada, Baetiscidae were later found in the fossil record as well, specifically in Cretaceous of Brazil and Eocene Baltic amber. An enigmatic piece of fossil evidence are two larvae from the Early Cretaceous Koonwarra Fossil Bed in Australia, which have been presumed as attributable to Baetiscidae and briefly discussed in previous studies. In the present contribution, we reinvestigate these fossils and confirm their attribution to the family Baetiscidae. These larvae are depicted and described as *Koonwarrabaetisca jelli* **gen. et sp. nov.** and *Koonwarrabaetisca duncani* **sp. nov.** For both Cretaceous genera *Protobaetisca* Staniczek, 2007 and *Koonwarrabaetisca* **gen. nov.** we establish a new subfamily Protobaetiscinae **subfam. nov.** within the family Baetiscidae, based on the presence of markedly shortened thoracic sterna. The phylogenetic position of newly described subfamily is clarified using a cladistic analysis; Protobaetiscinae **subfam. nov.** forms a monophyletic clade, sister to Baetiscinae. The confirmation of the distribution of Baetiscidae in the Cretaceous of Australia suggests almost worldwide distribution of this family in the deep time. Given their limited dispersal abilities, this distributional pattern can be best explained by the Pangean origin for this family, moving the time of their origin at least to the Early Jurassic. The larvae of *Koonwarrabaetisca* **gen. nov.** exhibit the same ecomorphological specialization as the rest of Baetiscidae, that supporting with a high probability their lifestyle similar to extant *Baetisca* Walsh, 1862. The larvae probably lived in the flowing water with stony substrate densely covered by filamentous algae, and in the places of accumulation of dead plant and algae matter during the last instars. Thus, *Koonwarrabaetisca* **gen. nov.** could be the allochthonous component in mayfly fauna of the Koonwarra paleolake.

## Introduction

The Koonwarra Fossil Bed in Australia represents one of the most prolific Mesozoic localities of fossil insects in the Southern Hemisphere. Thousands of specimens have been discovered, with the emphasis on immature stages^1^. At the same time, many specimens exhibit an excellent level of preservation, making Koonwarra one of the few Konservat-Lagerstätten in Australia (i.e., a sedimentary deposit with unusual occurrences and exceptional preservation of fossilized organisms or their traces)^2,3^. Among various insect taxa, mayflies (Ephemeroptera) are fairly abundant, with three described species^1,4^.

Further two fossil mayfly specimens from Koonwarra (each consisting of part and counterpart) were figured in Jell and Duncan^1^, who provided their morphological description without formal assignment to any species. Fossils were treated only as "Siphlonuridae? gen. nov." and listed as such also in the catalogue of Australian fossil insects, compiled in Jell^4^. The similarity of this material to the extant mayfly genus *Baetisca* Walsh, 1862 from the family Baetiscidae Edmunds & Traver, 1954 was noted already by Jell and Duncan^1^, however they considered attribution of these Australian fossils to Baetiscidae unlikely given the fact that extant distribution of this family is restricted to the North America.

The fossil record of Baetiscidae is relatively scarce yet attests to much wider geographical distribution in the past. Known Baetiscidae fossils were recently summarized in Staniczek et al.^5^ and contain four described species originating from the Cretaceous of Brazil and Eocene ambers in Europe and Russia. Therefore, its occurrence in the Cretaceous of Australia cannot be ruled out.

The mayflies of the family Baetiscidae are renowned for its charismatic larvae, equipped with greatly enlarged mesonotum forming a shield covering the thorax and most abdominal segments, usually possessing large spines with a protective function^6^. Phylogenetically, Baetiscidae together with Prosopistomatidae Lameere, 1917 and Cretomytarcyidae McCafferty, 2004 constitute the superfamily Prosopistomatoidea Lameere, 1917^5,7^. *Baetisca*, the only extant genus in the family, is endemic to North America, occurring over most of the eastern part of the continent to the Great Plains and the Northwest Territories of Canada^6^.

Regarding two fossil specimens of "Siphlonuridae? gen. nov." from Koonwarra, their similarity to *Baetisca* was mentioned not only by Jell and Duncan^1^, but also by Kluge^7^, who remarked upon the possible attribution to Posteritorna (which corresponds to Baetiscoidea Peters & Hubbard, 1989 or Carapacea, terms used by other authors, basically a group containing families Baetiscidae and Prosopistomatidae). Staniczek^8^ considered "Siphlonuridae? gen. nov." to represent either Baetiscidae or Baetiscoidea. Pescador et al.^9^ remarked about the presence of prominent transverse subapical ridge on the carapace, visible in the figure published in Jell and Duncan^1^. Based on this character, Pescador et al.^9^ hypothesized about the fossils to be closely allied to the stemline of Baetiscidae rather than Prosopistomatidae, the second family within Baetiscoidea. Staniczek et al.^5^ considered both specimens of "Siphlonuridae? gen. nov." from Koonwarra to be attributable to Baetiscidae, although not incorporating them into the phylogenetic analysis performed due to the lack of characters available.

As demonstrated above, those two fossils from Koonwarra have been repeatedly mentioned in practically all Baetiscidae-related papers since their original description, yet never studied again. All interpretations relied only on photographs published in Jell and Duncan^1^ and Jell^4^.

Therefore, we visited the Museum Victoria in Melbourne in 2022 and studied all material of the Koonwarra mayflies, including "Siphlonuridae? gen. nov.", presumed Baetiscidae specimens. Based on our investigation of this material, we supplement the information provided in Jell and Duncan^1^ and Jell^4^ and provide a new interpretation of those fossils. We also formally describe the material as two new species within the newly established genus *Koonwarrabaetisca* **gen. nov.** and discuss consequences for the phylogeny and historical biogeography of Baetiscidae.

## Results

### Systematic palaeontology

Class Insecta Linnaeus, 1758

Subclass Pterygota Lang, 1888

Order Ephemeroptera Hyatt & Arms, 1891

Family Baetiscidae Edmunds & Traver, 1954

Subfamily Protobaetiscinae **subfam. nov.**

LSID urn:lsid:zoobank.org:act:803C9003-01D7-405D-B30A-922C24852F0D

#### Type genus

*Protobaetisca* Staniczek, 2007

#### Type species

*Protobaetisca bechlyi* Staniczek, 2007 [by monotypy]

#### Genera included

*Protobaetisca* Staniczek, 2007 [Early Cretaceous, Crato Formation, Araripe Basin, Brazil]; *Koonwarrabaetisca* **gen. nov.** [Early Cretaceous, Koonwarra Fossil Bed, Australia].

#### Diagnosis

***Adult*** (modified from Staniczek et al.^5^ for putative adult of *P. bechlyi*): (**i**) body of small size, approx. 7 mm in length; (**ii**) thoracic sterna shortened, as long as approx. 0.20× body length; (**iii**) forewing length of approximately 6 mm, maximum width 3.5 mm; (**iv**) forewing triangular-shaped, width/length ratio approximately 0.58; (**v**) at least 15 simple and forked cross veins in pterostigma; (**vi**) MA slightly asymmetrical, fork located at 0.65 of its length; (**vii**) CuP nearly parallel to A1, situated close to each other; (**viii**) at least five veins going from A1 to basitornal margin; (**ix**) hind wings rounded, small, with prominent costal projection at base; ratio of hind/forewing length approx. 0.18; (**x**) abdominal segment VI largest. ***Mature larva*** (modified from Staniczek^8^ and Staniczek et al.^5^): (**xi**) body length 6.40–8.00 mm without caudal filaments; caudal filaments length 1.90–3.00 mm; (**xii**) genal shelf poorly developed, less indistinctly protruding above anterolateral margin of head; (**xiii**) thoracic sterna shortened, as long as 0.22× body length; (**xiv**) legs bicoloured; (**xv**) abdominal segments VI–VIII without prominent posterolateral projections and median spines.

- Genus *Protobaetisca* Staniczek, 2007

#### Type species

*Protobaetisca bechlyi* Staniczek, 2007 [by monotypy] in Staniczek^8^: 182.

#### Specimens included

*Protobaetisca bechlyi* Staniczek, 2007 [type species; larva, holotype SMNS 66620]; *P. bechlyi* Staniczek, 2007 [adult of unknown sex; SMF VI993].

#### Type locality, age and horizon

Vicinity of Nova Olinda, southern Ceará state, northeast Brazil; upper Aptian (approx. 113 Ma), Early Cretaceous, Nova Olinda Member, Crato Formation, Santana Group, Araripe Basin.

#### Revised diagnosis

As for type species, since monotypic (see below).

- *Protobaetisca bechlyi* Staniczek, 2007

= *Protobaetisca bechlyi* Staniczek, 2007; Staniczek^8^: *The Crato Fossil Beds of Brazil: window into an ancient world*, page 182 [original description]; Pescador et al.^9^: *Aquatic Insects*, page 139 [record, cladistic analysis]; Staniczek et al.^5^: *Arthropod Systematics & Phylogeny*, page 400 [redescription, first description of putative adult, phylogenetic analysis]

#### Revised diagnosis

(Supplementary Information 1, Fig. S3c). Modified from^8^ and^5^, with newly described characters of male larva (holotype) of *P. bechlyi*.

***Adult*** (as for Protobaetiscinae **subfam. nov.**; see above). ***Mature larva***: (**i**) body length approx. 8.00 mm (without caudal filaments), caudal filaments length approx. 3.00 mm; (**ii**) genal shelf moderately projected; (**iii**) antenna distinctly elongated; (**iv**) thoracic sterna short, as long as approx. 0.20× body length; prosternum/mesosternum/metasternum length ratio 1.00/0.90/0.50; (**v**) foreleg with femur short, as long as 0.32× tibia length; tibia longest, slender; tarsus at least 0.50× tibia length; preserved part of pretarsal claw at least 0.34× tibia length; (**vi**) notal shield robust, at least 0.75× as long as wide, expanded laterally, trace of putative lateral spine at left side; this lateral spine short and robust, at least 2.30× longer than wide; (**vii**) abdominal segments VI–VIII with prominent posterolateral projections; (**viii**) abdominal segment VI distinctly larger than segment VII [all as preserved].

#### Remarks

Recently, Staniczek et al.^5^ described a putative winged stage of *P. bechlyi*, specifying the presence of distinguishing characters of Baetiscidae, namely (**i**) longitudinal venation of forewing of "posteritornous" condition (i.e. CuP and A1 end distally of the forewing tornus); (**ii**) hind wing rounded, almost circular appearance; (**iii**) putative sharply pointed projection on ventral side of prothorax.

The revised diagnosis and redescription of the larva has been also proposed in Staniczek et al.^5^, based on a more detailed study of the holotype in comparison to original description published by Staniczek^8^. It was confirmed that the compressed specimen is visible from its ventral side, and a set of new characters of head (mouthparts), thorax and the tip of abdomen was described for the first time. The authors also reported about short mesosternum and metasternum, in contrast to relatively wide prosternum. On the other hand, as the larva is visible from its ventral side, other details of the thorax structure, especially the notal shield, have not been described. The present thorough reinvestigation of the holotype suggests the presence of a putative trace of lateral spine on the left side of notal shield and specifies its presumed shape and size (for more details see Fig. S3c and Table 1).

**Table 1:** Summary of generic adult and larval characters of extant and extinct Baetiscidae (modified based on previous authors^5,9,13,16^ and newly described characters). Distinct differential characters are marked in bold.

Characters	*Koonwarrabaetisca* gen. nov.	*Protobaetisca* Staniczek, 2007	*Balticobaetisca* Staniczek & Bechly, 2002	*Baetisca* Walsh, 1862 (incl. subgenera *Fascioculus* and *Baetisca* s.str.)
	Cretaceous Aptian 116 ± 5 Ma	Cretaceous Aptian approx. 113 Ma	Eocene Lutetian 34–48 Ma	Extant Nearctic realm
Adult	Adults unknown	Sex unknown	Male/female	Male/female
* Measurements*				
Body length [mm]	–	7.00*	6.80–9.75/8.50	5.00–12.0/5.50–12.30^1^
Forewings length [mm]	–	6.00*	8.30–11.75/9.80–10.10	6.00–12.8/8.00–14.50^1^
Hind wings length [mm]	–	3.50*	2.30–3.60/3.50–3.55	2.00–4.30/3.00–3.80^1^
** Forewings [width/length ratio]**	–	0.58*	**0.42–0.44**	**0.33–0.38**
**Hind/Fore wings length ratio**	–	0.18*	**0.26–0.31/0.35**	**0.30–0.34/0.26–0.37**
Hind wings [width/length ratio]	–	0.85*	0.74–0.80/0.78	0.72–0.86
*Head*				
Eyes [shape and structure]	–	–	Large, contiguous medially or separated by narrow gap/small, well separated	Large, contiguous medially or separated by narrow gap/small, well separated
Eyes [vertical bands]	–	–	Absent	Almost absent^4^
*Thorax*				
Prosternum [prominent bispinate projection]	–	? Present	Present	Present
Mesonotal suture [scutellum]	–	? Elongate	Elongate	Elongate
Mesothorax [anepisternum]	–	–	Distinctly smaller than katepisternum	Distinctly smaller than katepisternum
Mesothorax [furcasternal protuberances]	–	Elongate	Elongate, contiguous	Elongate, contiguous
**Thoracic sterna/body length ratio**	–	0.20*	**0.32–0.36/0.30**	**0.32–0.38**
* Forewing*				
Wings [colour]	–	–	Opaque, not translucent^2^/hyaline translucent^3^; no maculation	Hyaline or flushed with orange to reddish-brown (bands/maculation)
**Wings [shape]**	–	**Triangular, relatively wide**	**Moderately narrow**	**Moderately narrow**
Pterostigma [number of cross veins]	–	15*	8–13/15	8–14
Pterostigma [shape of veins]	–	Simple	Simple/mainly branched	Mainly simple
**RS furcation [respectively to vein length]**	–	0.30*	**0.13/0.18**	**0.14–0.18**
** MA furcation [respectively to vein length]**	–	0.65*	**0.56–0.58/0.54**	**0.52–0.56**
CuP and A1 [centrally]	–	Nearly parallel	Nearly parallel/divergent	Nearly parallel
**A1 [number of veins arising to basitornal margin]**	–	5*	**5–7/8–11**	**6–8**
A1 [shape of veins arising to basitornal margin]	–	Simple	Simple and forked veins	Simple and forked veins
A1–A2 [number of cross veins]	–	–	3–5/0	2–4
Intercalary veins between A1 and A2	–	–	0–2/6–10	0–1
Intercalary veins between A2 and A3	–	–	1–2/absent	Absent
*Hind wing*				
Wings [shape]	–	Nearly round	Nearly round	Nearly round
Costal projection [shape]	–	Blunt, prominent, situated at some distance from wing base	Blunt, prominent or slightly protruded, situated strongly proximally	Blunt, slightly protruded, situated strongly proximally
C–Sc field [number of cross veins in costal area]	–	–	4–12/5–8	5–10
C–Sc field [shape of cross veins in costal area]	–	–	Simple and forked	Simple and forked
C–Sc field [intercalary veins]	–	–	Almost absent	Almost absent
Free short marginal intercalaries	–	Present	Present	Present
*Legs*				
Foretarsi [shape of claws]	–	–	Both blunt/dissimilar (one blunt, one pointed)	Both blunt/dissimilar (one blunt, one pointed)
*Abdomen*				
Mid-dorsal transverse evaluation of tergum VI	–	–	Present	Present
Segment VI	–	Largest	Largest	Largest
**Sternum IX [in female]**	–	–	**Without apical cleft**	**Deeply cleft apically**
Paracercus		Vestigial	Vestigial	Vestigial
*Genitalia [male]*				
Forceps segment I [inner projection]	–	–	Triangular or widely rounded	Widely rounded
Forceps segment II [shape]	–	–	Nearly triangular or conical	Nearly triangular
** Penis lobes [shape]**	–	–	**Well separated, blunt apically**	**Fused, bluntly pointed to rounded apically**
**Larva**	**Female**	**Male**	**Larva unknown**	**Male/female**
*Measurements*				
Body length [without terminal filaments; mm]	6.40–6.60*	8.00*	–	6.00–14.00
Length of cerci [mm]	1.75–1.90*	3.00*	–	1.80–3.50
*Head*				
** Width/length ratio**	0.60–0.62*	0.45*	–	**0.48–0.64**
**Genal shelf**	**Not distinctly projected**	**Moderately projected, rounded apically**	–	**Well or moderately projected, rounded or sharp apically**
Frontal projection	Moderately projected, rounded apically*	Well projected, relatively large, rounded apically	–	Well projected, relatively large, rounded apically
Antennae/head length ratio	0.36*	0.68*	–	0.40–0.60
** Labrum**	**Nearly square**	–	–	**Rectangular**
*Thorax*				
**Thoracic sterna/body length ratio**	0.22*	0.20*	–	**0.31–0.36**
**Prosternum/mesosternum/metasternum length ratio**	1.00/0.80–0.90/1.30–1.60*	1.00/0.90/0.50*	–	**1.00/1.80–2.50/0.90–1.55**
** Prothorax [laterally]**	**Moderately wide; well separated from mesothorax**	–	–	**Relatively narrow; not distinctly separated from mesothorax**
Prosternum [width/length ratio]	–	0.30*	–	0.24–0.28
Mesonotal shield [shape]	Tall, dorsoventrally not compressed	–	–	Tall, dorsoventrally not compressed or dorsoventrally moderately compressed
** Notal shield [length/width ratio; without lateral spines]**	–	0.75*	–	**0.82–1.50**
Lateral spines	? Weakly developed [lateral aspect]*	? Developed	–	Well developed
** Lateral spine [length/width ratio]**^5^	–	2.30*^6^	–	**1.00–2.00**
Medial hump	Weakly developed [lateral aspect]*	–	–	Absent or prominent
Dorsal projections	? Weakly developed [lateral aspect]*	–	–	Absent or present; if present, well or weakly developed
**Legs [colouration]**	**Bicoloured**	**Bicoloured**	–	**Unicoloured**
**Forelegs [femur/tibia/tarsus/pretarsus length ratio]**	1.60–1.90/1.00/0.80*–0.96*^7^	0.32/1.00/0.50/0.34*	–	**1.85–2.10/1.00/1.34–1.48/0.92–1.20**
** Foretibia [shape]**	**Short, stout, expanded centrally**	**Long, slender along the entire length**	–	**Short, stout, expanded centrally**
*Abdomen*				
Abdominal segment VI [size respectively to segments V and VII]	Equal or slightly larger	Distinctly larger	–	Distinctly larger
**Abdominal segments VI–VIII [posterolateral projections]**	**Absent**	**Present, prominent**	–	**Present, prominent or moderately prominent**
**Abdominal segments VI–IX [posteromedian elevations]**	**Absent**	–	–	**Present, prominent**
**Abdominal segment IX [posterolateral projections]**	**Present or absent**	**Present, prominent**	–	**Present, prominent**

*Remarks*: ^1^Based on original data and Pescador and Berner^13^.

^2^In male subimago (see Staniczek et al.^5^).

^3^In female imago (see Staniczek and Bechly^16^).

^4^Present in a single species *Baetisca* (*Fascioculus*) *escambiensis* Berner, 1955 (see Kluge^7^ and Pescador and Berner^13^).

^5^The ratio is measured as the maximal length to the width at base of lateral spine (see Pescador and Berner^13^).

^6^Putative trace of lateral spine is marked by dashed line on the left side of notal shield of *Protobaetisca bechlyi* Staniczek, 2007.

^7^Measured only for the holotype larva of *Koonwarrabaetisca duncani* **sp. nov.**

*As preserved.

- Genus *Koonwarrabaetisca* **gen. nov.**
- LSID urn:lsid:zoobank.org:act:1C668D0E-9C7C-4476-BEED-0045A7D1A4EC

 = Siphlonuridae? gen. nov. in^1^: *Mem. Ass. Australasian Palaeontology*, pages 118, 119, 126 [description]; Jell^4^: *Memoirs of the Queensland Museum*, page 12, unnumbered figure and photo [record, short description]; Kluge^7^: *The Phylogenetic System of Ephemeroptera*, page 63 [similarities with Baetiscidae; possible placement within Prosopistomatoidea]; Staniczek^8^: *Ephemeroptera: mayflies*, page 183 [stem group of Baetiscidae or Prosopistomatoidea]; Pescador et al.^9^: *Aquatic Insects*, page 139 [placement in the stem line of Baetiscidae; cladistic analysis]; Staniczek et al.^5^: *Arthropod Systematics & Phylogeny*, page 405 [placement in Baetiscidae; new diagnostic characters]

#### Type species

*Koonwarrabaetisca jelli* **sp. nov.**

#### Species included

*Koonwarrabaetisca jelli* **sp. nov.**, *K. duncani* **sp. nov.**

#### Specimens included

See “Material and methods”.

#### Type locality, age and horizon

Vicinity of Koonwarra (3 km east), South Gippsland, Highway near Tarwin, Victoria, Australia; middle–late Aptian (118 ± 5–115 ± 6 Ma), Early Cretaceous, Koonwarra Fossil Bed, Korumburra Group.

#### Diagnosis

***Mature larva***: (**i**) body length 6.40–6.60 mm (without caudal filaments); (**ii**) genal shelf not distinctly projected; (**iii**) antenna relatively short; (**iv**) thoracic sterna short, as long as approx. 0.22× body length; prosternum/mesosternum/metasternum length ratio 1.00/0.66–0.80/1.36–1.60; (**v**) foreleg with femur long, approx. 1.60–1.90× tibia length; tibia short, stout, expanded apically; tarsus app. 0.80–0.96× tibia length; (**vi**) mesonotal shield robust, not compressed dorsoventrally; lateral spine weakly developed (visible in lateral aspect); (**vii**) abdominal segments VI–VIII without posterolateral projections; (**viii**) abdominal segment VI equal or slightly larger than segment V and VII [all as preserved].

Adults unknown.

#### Derivation of name

The generic name of female gender is a composition of the "Koonwarra", the fossil bed where both larvae attributed to this genus were collected, combined with the extant genus *Baetisca*.

#### Remarks

When describing the structure of the thorax of *Koonwarrabaetisca* **gen. nov.**, we use the term "mesonotal shield" to characterise the unique structure of larval thoracic terga. In all studied imprints of *Koonwarrabaetisca* **gen. nov.**, a more or less distinct boundary between pronotum and mesonotum is visible. The larva *Protobaetisca* is accessible for examination from the ventral side only, so the boundary between pronotum and mesonotum is not possible to observe. In extant *Baetisca* the margin between pronotum and mesonotum is visible as a prominent crest covered by dense row of tubercles. On the other hand, the margin between pronotum and mesonotum in *Koonwarrabaetisca* **gen. nov.** is apparent as shallow fold in both species. Since the level of separation of pronotum and mesonotum in *Koonwarrabaetisca* **gen. nov.** seems more pronounced compared to *Baetisca*, we prefer to use here the term "mesonotal shield" instead of "notal shield", which incorporates also pronotum as its integral part.

- *Koonwarrabaetisca jelli* **sp. nov.**
- LSID urn:lsid:zoobank.org:act:791875F7-F1D2-4DC8-A133-8B574CC640B2

**Figure 1:**
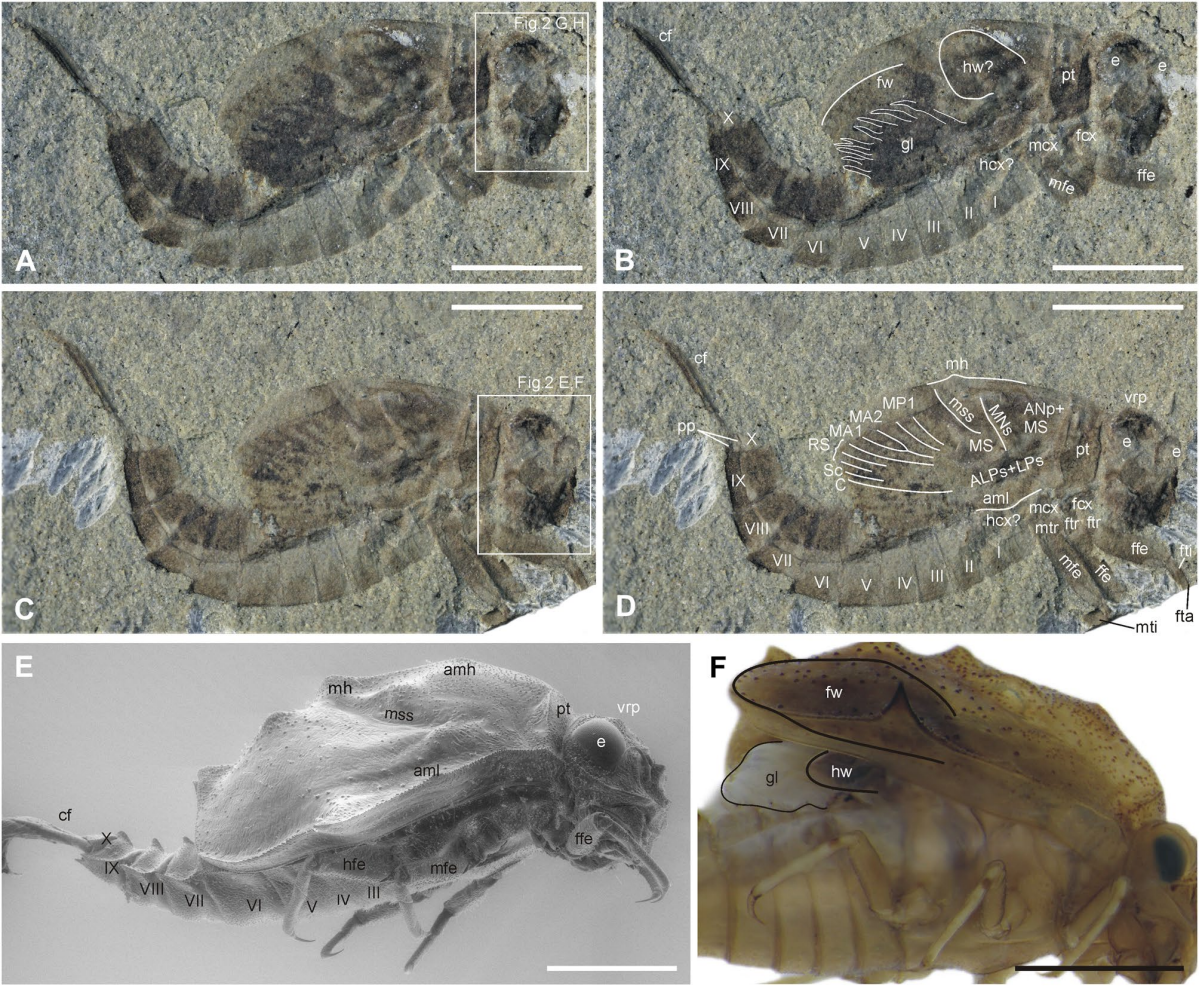
*Koonwarrabaetisca jelli* **gen. et sp. nov.** (**A**–**D**) and comparative Recent material of *Baetisca rogersi* Berner, 1940 (**E**–**F**). (**A**) Specimen P103210A, general view; (**B**) Specimen P103210A, general view with interpretation of individual body parts; (**C**) Specimen P103210B, general view; (**D**) Specimen P103210B, general view with interpretation of individual body parts; (**E**) *Baetisca rogersi*, general view laterally under ESEM; (**F**) *Baetisca* sp., position of tracheal gills and hind wing pad. Scale bar = 2 mm (**A**–**F**). Rectangles in figures A and C mark positions of detailed figures. Figures C and D are mirror inverted. Abbreviations. *Head*: e—eye; vrp—vertex protuberance; *Thorax*: pt—prothorax; amh—anteromedian hump; mh—medial hump; aml—anteromedian lobe; MNs—mesonotal suture; ALPs + LPs—anterolateroparapsidal suture and lateroparapsidal suture; ANp + MS—anteronotal protuberance and medioscutum; mss—medioscutal suture; fw—forewing pad [white or black line indicates the placement of tornoapical margin]; hw—hind wing pad [white or black line indicates the shape of putative hind wing remnants]; fcx—forecoxa; ftr—foretrochanter; ffe—forefemur; fti—foretibia; fta—foretarsus; mcx—middle coxa; mtr—middle trochanter; mfe—middle femur; mti—middle tibia; hcx—hind coxa; *Abdomen*: I–X—abdominal segments I–X; gl—tracheal gills [white or black line indicates the placement of putative tracheal gills]; pp—paraproct plates; cf—caudal filaments. Interpretation of larval wing pad venation is indicated by white lines; for vein abbreviations see Kluge^7^.

**Figure 2:**
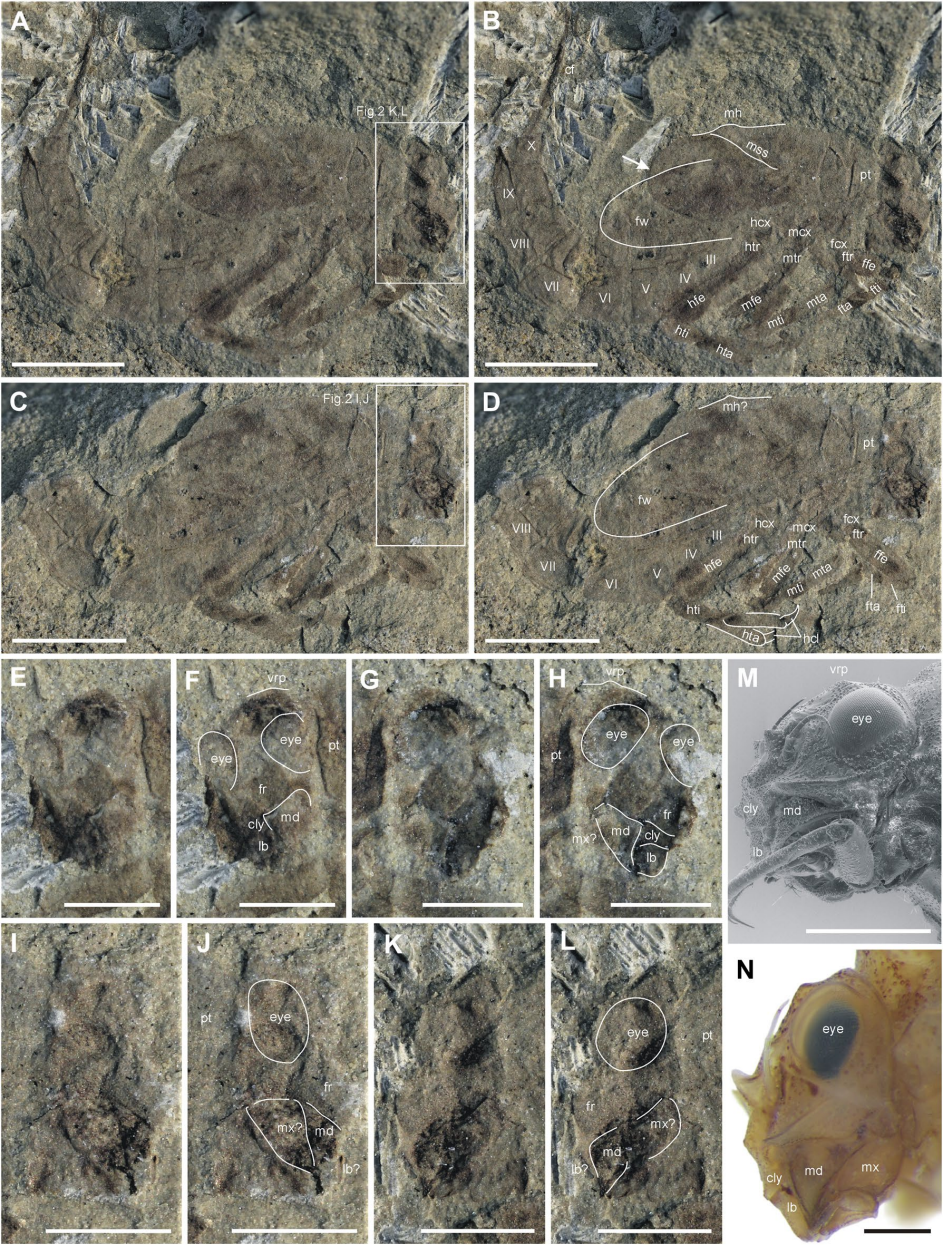
*Koonwarrabaetisca duncani* **gen. et sp. nov.** (**A**–**L**) and comparative Recent material of *Baetisca rogersi* Berner, 1940 (**M**, **N**). (**A**) Specimen P103209A, general view; (**B**) Specimen P103209A, general view with interpretation of individual body parts; (**C**) Specimen P103209B, general view; (**D**) Specimen P103209B, general view with interpretation of individual body parts; (**E**) Specimen P103210B, head; (**F**) Specimen P103210B, head with interpretation of individual body parts; (**G**) Specimen P103210A, head; (**H**) Specimen P103210A, head with interpretation of individual body parts; (**I**) Specimen P103209B , head; (**J**) Specimen P103209B, head with interpretation of individual body parts; (**K**) Specimen P103209A , head; (**L**) Specimen P103209A, head with interpretation of individual body parts; (**M**) *Baetisca rogersi*, head laterally under ESEM; (**N**) *Baetisca rogersi*, head laterally. Scale bars = 2 mm (**A**–**D**) and 1 mm (**E**–**N**). Rectangles in figures A and C mark positions of detailed figures. Figures A and B are mirror inverted. Abbreviations. *Head*: cly—clypeus; fr—frons; frp—frontal protuberance; lb—labrum; md—mandible; mx—maxilla; vrp—vertex protuberance [white lines in Figures F, H, J and L indicate the shape of eyes and borders between respective mouthparts]; *Thorax*: pt—prothorax; mh—medial hump; mss—medioscutal suture; fw—forewing pad [white or black line indicates the shape of the wing]; fcx—forecoxa; ftr—foretrochanter; ffe—forefemur; fti—foretibia; fta—foretarsus; mcx—middle coxa; mtr—middle trochanter; mfe—middle femur; mti—middle tibia; mta—middle tarsus; hcx—hind coxa; htr—hind trochanter; hfe—hind femur; hti—hind tibia; hta—hind tarsus; hcl—hind claw; *Abdomen*: I–X—abdominal segments I–X; cf—caudal filaments.

Figures 1A–D, 2E–H, S1a, c, S3a, b; Table 1.

Siphlonuridae? gen. nov. in Jell and Duncan^1^: *Mem. Ass. Australasian Palaeontology*, pages 118, 119, 126 [description]; Jell^4^: *Memoirs of the Queensland Museum*, page 12, unnumbered figure and photo [record, short description]

#### Type material

***Holotype.*** Mature female larva, NMVP103210 (part [A] and counterpart [B]), Museum Victoria collection (Melbourne, Australia).

#### Type locality, age and horizon

See above.

#### Derivation of name

The species is named in honour of Peter A. Jell, famous Australian palaeontologist who discovered and described the freshwater invertebrates from the Early Cretaceous from the Koonwarra fossil record, including mentioned specimen.

#### Diagnosis

(**i**) Body length ca 6.40 (without caudal filaments); (**ii**) Putative remnants of lateromedian lobe and lateral spine of mesonotal shield are small and not protruded; (**iii**) No posterolateral projection on abdominal segment IX.

#### Description

*Generalities*. Specimen is preserved in lateral view, with body almost complete; part of legs and distal end of mesonotal shield are missing; antennae incomplete, poorly preserved; the shape of possible remnants of hind wing and gills are recognisable in part [A], while structure of forewing with traces of longitudinal venation is preserved on counterpart [B]. No traces of setation and tubercles on surface of body.

Length of body without terminal filaments 6.40 mm; length of preserved part of cerci 1.90 mm. Head, thorax and abdominal terga mainly darker than abdominal sterna (Figs. 1A–D, S3a, b).

#### Head

Poorly preserved, hypognathous, not wide, slightly rotated frontolaterally; head width at least 0.60× head length. Compound eyes well visible, large, closely converge medially, approximately 1.10–1.30× longer than wide. Head cuticle without traces of pronounced corrugation or tubercles on surface. Epicranial suture poorly preserved, relatively narrow, nearly Y-shaped. Clypeus relatively short and narrow, not expanded laterally. Antennae not clearly distinguishable, partly preserved, seem to be short, as long as at least 0.36× head length; putative remnants of basal antennal segment poorly visible. Genal shelf not distinctly projected. Frontal projection rounded apically, moderately projected. Labrum nearly square, slightly elongated, larger than clypeus; outer margin of labrum smoothly rounded. Remnants of mandible large, well visible, slightly curved inwards distally; apical denticulation of mandible poorly visible; putative incisors strong and elongated; no traces of preserved tubercles on surface of mandible. Maxillary stipes elongated, apically terminated with strong canine. Other mouthparts not distinguishable (Figs. 2E–H, S1a, c).

#### Thorax

Laterally compressed, almost complete except distal end; remnants of forewing better visible on NMVP103210A, possible remnants of hind wing and tracheal gills visible on NMVP103210B.Thorax relatively tall. Prothorax moderately wide; margins well defined; anterior margin distinct; margin between prothorax and mesonotal shield well visible on NMVP103210A as a thin line; on NMVP103210B posterior margin preserved as shallow fold, possibly as a result of preparation; prominent carina along anterior and posterior margins of pronotum; no other trace of humps and projections on prothorax.

Mesonotal shield moderately tall, covers metathorax and part of abdomen, distally up to middle of abdominal segment VI; meso- and metanotum fused, at least no visible separation between these segments. Dorsal side of mesonotal shield not flattened or compressed, moderately dome-shaped. Mesonotal humps and projections not prominent, possibly as a result of lateral compression; medial hump and putative remnants of dorsal projection moderately protruded; no traces of anteromedian lobe; lateromedian lobe and putative remnants of lateral spine small, not protruded.

Thoracic sterna distinctly short, 0.22× as long as body length; mesosternum as long as 0.90× prosternum; metasternum partly damaged, not shortened (see Table 1).

Remnants of adult mesonotum and its sutures partly visible inside mesonotal shield. Fore- and hind wing pads partly preserved. Forewing elongated and relatively narrow; vein precursors well visible in their distal part; all triads seem to be developed; numerous longitudinal veins in RS; MA fork nearly symmetrical; putative CuP and A1 seem to be situated after of tornus. Possible remnants of hind wing nearly round, shifted anteriorly and dorsad, poorly visible basally, venation unclear.

Legs poorly preserved, lacking all tarsi, pretarsal claws and hind legs (except incomplete, putative hind coxa); only proximal part of fore- and middle tibia preserved. Forefemora shortest, relatively stout, approximately 3.25× longer than wide; middle femur longer, with length/width ratio 4.38; diffuse dark macula on femora proximally and centrally (Figs. 1A–D, S3a, b).

#### Abdomen

All segments relatively well-preserved, almost complete, terga and sterna visible in lateral aspect, moderately tall; no posterolateral projections on abdominal segments VI–IX; no preserved traces of posteromedian elevations on terga dorsally; abdominal segment VI not enlarged, similar in size to segment VII, and seems equal or slightly larger that segment V; segment IX slightly elongated; no traces of posterolateral projection on segment IX; tergite X small, triangular-shaped in lateral aspect; paraprocts slightly elongated, bluntly pointed apically; visible part of sternum I shortest.

Contours of six gill plates are visible inside of mesonotal shield, their structure and shape not distinguishable in detail; the size of plates seems to slightly decrease from first to last plate.

Cerci and paracercus stout, nearly subequal in length; long swimming setae on both inner sides of cerci and paracercus, denser at mid-length; lacking on proximal 1/4 of caudal filaments length (Figs. 1A–D, S3a, b).

- *Koonwarrabaetisca duncani* **sp. nov.**
- LSID urn:lsid:zoobank.org:act:DA13070E-BA23-4A59-9516-A41F10FB7730

Figures 2A–D, I–L, S2a, d, S4a, c; Table 1.

Siphlonuridae? gen. nov. in Jell and Duncan^1^: *Mem. Ass. Australasian Palaeontology*, pages 118, 119, 126 [description]

##### Type material

***Holotype.*** Mature female larva, NMVP103209 (part [A] and counterpart [B]), Museum Victoria collection (Melbourne, Australia).

##### Type locality, age and horizon

See above.

##### Derivation of name

We dedicate this species to Peter M. Duncan, who first discovered and described together with Peter A. Jell the larvae of *Koonwarrabaetisca* **gen. nov.** in the Early Cretaceous of Australia.

##### Diagnosis

(**i**) Body length ca 6.60 (without caudal filaments); (**ii**) Lateromedian lobe and lateral spine of mesonotal shield are moderately protruded; (**iii**) Prominent posterolateral projections on abdominal segment IX.

##### Description

*Generalities*. Specimen is preserved in lateral view; body almost complete except of legs. Distal portion of mesonotal shield seems shorter in part [A], covering distally abdominal segment V, which is probably a result of the preservation features (peculiarly shortened distal end of mesonotal shield is marked by white arrow on Fig. 2B). Abdominal segments VI and VII partly damaged; most part of abdominal segment IX, and entire segment X and cerci lost in part [A]. No visible setation and tubercles on surface of body, except of legs.

Length of body without terminal filaments 6.60 mm; length of preserved part of cerci 1.75 mm. Legs and part of mesonotal shield slightly darker than rest of body (Figs. 2A–D, S4a, c).

#### Head

Both preserved part and counterpart very damaged; head hypognathous, relatively narrow, visible in lateral aspect, at least 0.62× as wide as long; vertex seems slightly elongated, but without projections or hump. Compound eyes damaged, probably large, slightly elongated. No preserved traces of tubercles, setation, sutures and corrugation of surface of head. Clypeus relatively narrow, poorly preserved; no preserved traces of antennae. Genal shelf smoothly protruding, bluntly pointed apically. Frontal projection presumably rounded apically. Labrum partly preserved, nearly square-shaped, slightly elongated; outer margin of labrum smoothly rounded. Mandible and maxilla large, partly visible laterally; mandible with stout incisors distally; segmentation of putative maxillary palp poorly visible, presumably consists of three short segments nearly equal in length. Maxillary stipes elongated. Putative remnants of labium hardly distinguishable (Figs. 2I–L, S2a, d).

#### Thorax

Laterally compressed, almost complete; unclear trace of forewing pad inside of mesonotal shield, clearer on NMVP103209B, covering distally abdominal segment VI; no preserved remnants of gills inside of mesonotal shield. Thorax relatively tall. Prothorax relatively wide; anterior and posterior margins well distinguishable, without visible prominent carina; no preserved traces of marginal tubercles along of prothorax margins; pronotal-mesonotal elevation preserved as visible shallow fold; no other trace of humps and projections on prothorax.

Mesonotal shield dome-shaped, distal end damaged; meso- and metanotum fused; putative remnant of medial hump and dorsal projection relatively flat, thereby no prominent projections on dorsal side of mesonotal shield; no traces of anteromedian lobe; putative remnants of lateromedian lobe and lateral spine of mesonotal shield moderately protruded.

Thoracic sterna short, 0.22× as long as body length; mesosternum shortest, as long as 0.80× prosternum. Metanotum largest, up to 1.60× as long as prosternum length (see Table 1).

Remnants of adult mesonotum indistinctly visible inside of mesonotal shield, forewing pad relatively narrow, reaches posterior margin of abdominal segment VI [upper part of surrounding mesonotal shield lost]; forewing pad venation not preserved except of two unclear longitudinal veins, possibly belonging to RS field. Remnants of hind wing not visible. Legs slender and long, mostly preserved; diffuse dark maculae on femora, tibiae and tarsi. Femora long; tibiae and tarsi combined nearly equal to femora in length. Forefemora stout, slightly widened centrally, as long as 1.60–1.90× foretibiae, and nearly twice longer than foretarsi; forefemora approximately 3.25× as long as wide; middle and hind femora markedly longer, with length/width ratio 6.80–7.20. A few stout spatulate setae on femora accompanied with sparse short setae (well visible on left forefemur). Preserved pretarsal claws seem to be stout, short and moderately hooked (Figs. 2A–D, S4a, c).

#### Abdomen

All segments well preserved, almost complete except segments VI and VII that are partly damaged, but seem to be nearly equal in size; abdominal segments relatively tall; segment IX elongated, longer than segment VIII; prominent posterolateral projections on abdominal segment IX, reaches middle of segment X length; tergum X smallest; paraprocts elongated, triangular-shaped, bluntly pointed apically. Sternum I shortest; sterna II–IV equal in length, slightly shorter than sternum V. Two oblique dark smudges along abdominal segments I and II could be associated with putative remnants of gill plates (visible on counterpart NMVP103209B). Cerci and paracercus stout, distal parts missing; setation poorly visible on a few distal segments only (Figs. 2A–D, S4a, c).

## Discussion

### Taxonomy

#### The position of *Koonwarrabaetisca* gen. nov. in Posteritorna and Baetiscidae

**Figure 3:**
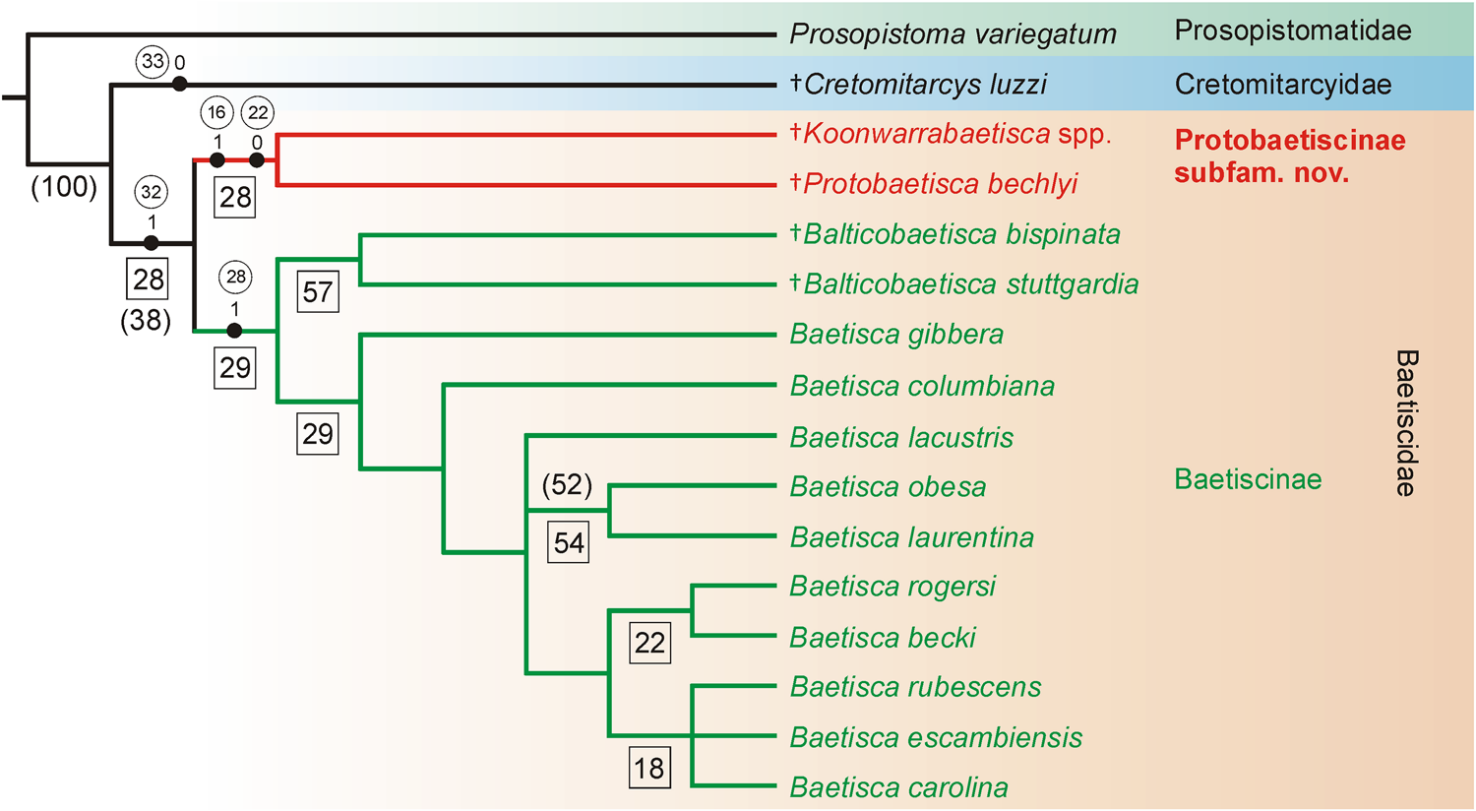
Strict consensus tree from the parsimony analysis of Recent and fossil taxa. Numbers in circles correspond to characters representing synapomorphies (numbering of characters as in the Table S1 and S2, presented only for basal nodes). Character states for individual branches are in black circles. Relative Bremer support values are located below branches, in black squares. Bootstrap support values are represented by numbers in brackets (only values higher than 30 are listed). Only adult males of the genus *Balticobaetisca* Staniczek & Bechly, 2002 are included to the analysis.

The larvae of *Koonwarrabaetisca* **gen. nov.** were mentioned for the first time by Jell and Duncan^1^, who attributed them as putative members of Siphlonuridae; this attribution was later followed in Jell^4^. Despite the questionable attribution, the first short description in Jell and Duncan^1^ was very precise. For example, the authors pointed out the structure of the head and mouthparts in details, with narrow clypeus and approximately square labrum rounded apically, large mandible, and preserved remnants of labium, all of which agree with our observations (Figs. 2E–H, I–L, S1a, c, S2a, d). The presence of prominent transversal folds along the anterior and posterior margins of pronotum was also reported (termed transverse carina), as well as large notal shield (mesonotum) expanded posteriorly, reaching abdominal segment V. In fact, the distal end of the notal shield in specimen NMVP103210 reaches at least the middle of abdominal tergite VI (Figs. 1, S3a, b). The authors also noted the presence of eight preserved longitudinal radiating veins depicted in Fig. 3E in^1^ for *K. jelli* **sp. nov.** (specimen NMVP103210), as well as the lack of posterolateral projections on abdominal segments, except segment IX in *K. duncani* **sp. nov.** (specimen NMVP103209).

Summarizing all current data and results of the original material investigation, we define the systematic position of *Koonwarrabaetisca* **gen. nov.** in Posteritorna and more precisely in Baetiscidae based on the combination of several characters.

(i) In *Koonwarrabaetisca* **gen. nov.** the preserved forewing pad is elongated and relatively narrow, with presumably posteritornous condition, wherein CuP and A1 seem to be situated behind tornoapical margin (tornus) of the wing, with traces of veins going from A1 to the tornus and basitornal margin. Furthermore, the MA and MP fields are narrow, MA fork nearly symmetrical, with bifurcation almost at half of MA length (Figs. 1D, S3b). Possible remnant of hind wing is nearly rounded, similarly to all extant and extinct taxa associated with Baetiscidae, in contrast to the rest of Posteritorna, where the hind wing is elongated (see Figs. 1B, S3a;^7,10–12^). However, in comparison with other Baetiscidae hind wing pad in *K. jelli* **sp. nov.** is uncharacteristically located, shifted anteriorly and dorsad, which is probably a feature of the specimen fossilization.
(ii) Except of the position of the forewing tornus, another apomorphy listed for Posteritorna is the structure of larval thorax modified to notal shield (Figs. 1E, F, S4b;^7,9^). In extant Baetiscidae, notal shield is formed by completely fused and strongly enlarged pronotum, mesonotum and forewing pads (or protoptera sensu Kluge^7^), with the margin between pronotum and mesonotum only visible as a prominent crest covered by dense row of tubercles ^7,13^. In *Koonwarrabaetisca* **gen. nov.**, larval pronotum is more distinctly separated from mesothorax by a suture (Figs. 1, 2, S3, S4). The margin between pronotum and mesonotum appears slightly different in two species of *Koonwarrabaetisca* **gen. nov.**, which could be related to the material fossilization and/or preparation. In *K. jelli* **sp. nov.** it is preserved as a longitudinal thin shallow furrow, whereas in *K. duncani* **sp. nov.** a less prominent crest is visible on NMVP103209A and a distinct furrow on NMVP103209B (Figs. 1, 2, S3, S4). We assume that the pronotum could have been partly separated from the mesonotum, providing larvae from Koonwarra with higher mobility of the thoracic articulation than in the extant Baetiscidae. Mesonotal shield of *Koonwarrabaetisca* **gen. nov.** covers abdominal terga I–VI similarly to extant Baetiscidae and forms a gill chamber^7^.
(iii) The presence and structure of gills of the *Koonwarrabaetisca* **gen. nov.** has not been previously reported^1,7^. We observed the presence of the contours of gill plates inside of a mesonotal shield (gill chamber), partly covered by the remnants of wing venation (Figs. 1A, B, S3a). The shape and location of the gill plates resemble those depicted for extant Baetiscidae (see Fig. 20A in ^14^), with the size of plates slightly decreasing from the first to the last one (Figs. 1A, B, F, S3a).

Additionally, we have found that abdominal segment VI in *K. jelli* **sp. nov.** is almost as large as the adjacent segments V and VII (Fig. 1A–D). Abdominal segment VI of *K. duncani* **sp. nov.** is partly damaged distally but seems to be the same size or slightly larger than neighbouring segments (Fig. 2A–D). This feature well distinguishes the new genus from *Baetisca* and *Protobaetisca*, which have the abdominal segment VI distinctly largest (Figs. 1E, S3c, S4b). The abdomen of both larvae of *Koonwarrabaetisca* **gen. nov.** were fossilized being bent dorsally. This bend slightly distorts segments VI to VIII, making their correct size difficult to measure.

Our findings are in agreement with earlier authors who suspected the attribution of *Koonwarrabaetisca* **gen. nov.** to Posteritorna or Baetiscidae^5,7–9^.

#### Justification for designation of Protobaetiscinae subfam. nov.

The extinct members of Baetiscidae with known larvae include two genera, *Protobaetisca* and *Koonwarrabaetisca* **gen. nov.** Both taxa share several morphological characters, separating them from all extant members of Baetiscidae.

Specifically, *Protobaetisca* and *Koonwarrabaetisca* **gen. nov.** exhibit markedly shortened thoracic sterna, which represent a unique character within Baetiscidae. The length of thoracic sterna reaches 0.20× (in *Protobaetisca*) to 0.22× (in *Koonwarrabaetisca* **gen. nov.**) body length, in contrast to extant Baetiscidae with the thoracic sterna to body length ratio 0.31–0.36, measured for all extant species. Furthermore, larval mesosternum of *Protobaetisca* and *Koonwarrabaetisca* **gen. nov.** is shorter than prosternum (see Table 1; Figs. 1, 2, S3, S4), whereas mesosternum is always longer in extant Baetiscidae (Figs. 1E, F, S4).

It should also be noted that the larval prothorax of *Protobaetisca* and *Koonwarrabaetisca* **gen. nov.** seems relatively wide and robust compared to extant species (Table 1). Recently, Staniczek et al.^5^ reported that thoracic sterna of larval stage of *Protobaetisca* are short, with prosternum well separated from head and anterior part of mesothorax.

In the cladistic analysis, Baetiscidae were supported as monophyletic and split into two clades, one consisting of *Koonwarrabaetisca* **gen. nov.** and *P. bechlyi*, the second with all extant representatives of Baetiscidae together with fossil species of *Balticobaetisca*. Thus, *Protobaetisca* and *Koonwarrabaetisca* **gen. nov.** formed a monophyletic group sister to all other Baetiscidae (Fig. 3). The traditional and new technology search gave the same topology in the deeper nodes; differences occurred only in the branching pattern between extant *Baetisca* species, not affecting the conclusions presented here. The branch support is low, attributable to the large number of unknown characters, which is to be expected in fossil species where only some life stages are known. Nevertheless, the *Protobaetisca* + *Koonwarrabaetisca* **gen. nov.** clade it is supported by two apomorphies, character 22 reflecting short thoracic sterna (see above) and character 16, presence of bicoloured legs, with darker bands observable on femora, tibiae and tarsi (Fig. 3). Our results are consistent with the earlier phylogeny presented in Staniczek et al.^5^. However, their cladogram did not contain specimens from Koonwarra, since they did not study the material and were unable to extract most characters from the earlier published figures and descriptions.

Based on the unique structure of thoracic segments and a distinct position within the Baetiscidae phylogeny, we include both fossil genera *Protobaetisca* and *Koonwarrabaetisca* **gen. nov.** into the newly established subfamily Protobaetiscinae **subfam. nov.** The adult characters defining the new subfamily are questionable, since only *Protobaetisca* is known in the adult stage, described by Staniczek et al.^5^ from the Early Cretaceous Crato Formation of Brazil. Similarly to the holotype larva, the alate specimen of *Protobaetisca* is characterized by shortened thoracic sterna, which are approximately 0.20× body length. This character is markedly differentiating adults of Protobaetiscinae **subfam. nov.** from Baetiscinae (including Eocene *Balticobaetisca*), with thoracic sterna/body length ratio 0.30–0.38 (Table 1). Additionally, adults of Protobaetiscinae **subfam. nov.** can be defined by (**i**) the relatively wide and triangular-shaped forewing (in contrast to narrower forewing in Baetiscinae); (**ii**) the placement of RS furcation distant 0.30 of vein base (0.13–0.18 in Baetiscinae); (**iii**) the placement of MA furcation distant at least 0.65 of its base (0.52–0.58 in Baetiscinae); (**iv**) the shape of CuP and A1, nearly parallel along all their length, and closely approximated to each other (in contrast to these veins situated far from each other in Baetiscinae); (**v**) anal veins of the forewing are unbranched and fewer in number in Protobaetiscinae **subfam. nov.** (Table 1 and Fig. 7 in Staniczek et al.^5^).

#### Differentiation between *Koonwarrabaetisca* gen. nov. and other Baetiscidae

The larvae of *Koonwarrabaetisca* **gen. nov.** differ from other baetiscid genera by the following combination of morphological characters: (**i**) genal shelf not distinctly projected (in contrast to moderately projected and apically rounded genal shelf in *Protobaetisca*, and well-projected, apically rounded or acute genal shelf in extant Baetiscidae); (**ii**) thoracic sterna shortened, as long as approximately 0.22 of body length similarly to the genus *Protobaetisca* [0.20] (in contrast to enlarged thoracic sterna of *Baetisca*); mesosternum shortest (in contrast to enlarged mesosternum in other Baetiscidae); (**iii**) the length ratio of foreleg segments, with longest femur (in contrast to *Protobaetisca*), and moderately developed tarsus (in contrast to other Baetiscidae); (**iv**) mesonotal shield robust, not compressed dorsoventrally (unlike most *Baetisca*); (**v**) and (**vi**) abdominal segments VI–VIII without posterolateral projections, and segment VI not largest (in contrast to other Baetiscidae) (Table 1).

In the taxonomy of Baetiscidae, an important character is the size and shape of various protuberances of the thorax. Both larvae of *Koonwarrabaetisca* **gen. nov.** are preserved laterally, with at most small-sized lateromedian lobe and especially lateral spine visible (Figs. 1, 2, S3a, b, S4a, c). The remnants of a median hump and dorsal projection are only poorly recognizable. Apparently, these projections were not damaged or deformed during the fossilisation, given that the fossil specimens are relatively well-preserved. The compression of larvae in lateral position could lead to damage of these projections, if they were large and protruding strongly beyond the edges of the mesonotal shield, which was not the case in *Koonwarrabaetisca* **gen. nov.** Nevertheless, when considering the relationship of *Koonwarrabaetisca* **gen. nov.** to other representatives of the family Baetiscidae, we use the shape, location and size of the projections on the mesonotal shield only with caution. The larva of the second fossil baetiscid genus *Protobaetisca* is known as holotype preserved ventrally. In both detailed descriptions of *P. bechlyi* there is information about lacking mesonotal shield projections^5,8^. However, our reinvestigation of the holotype of *P. bechlyi* suggests that poorly preserved remnants of the left lateral spine could be discernible, although covered by surrounding matrix. At least some remnants are visible at the place where lateral spine should be located (Fig. S3c).

#### Differences between two new species of *Koonwarrabaetisca* gen. nov.

We describe the Baetiscidae larvae that have been found in the Koonwarra Fossil Bed as two separate species, *K. jelli* **sp. nov.** and *K. duncani* **sp. nov.** The marked difference between these species can be found in the structure of abdominal segment IX, equipped with prominent posterolateral projection in *K. duncani* **sp. nov.**, whereas this projection is missing in *K. jelli* **sp. nov.** (Figs. 1A–D, 2A–D). Some minute differences can also be found on the structure of the middle femora, which seem to be longer and thinner in *K. duncani* **sp. nov.** (Table 1). These differences are not attributable to sexual dimorphism, since we consider that both specimens of *Koonwarrabaetisca* **gen. nov.** to represent mature larvae of females. The late instar of larval development is indicated by the wing pads with visible venation pattern. In contrast to *P. bechlyi* with preserved putative genital buds indicating the male, the tip of larval abdomen of *Koonwarrabaetisca* **gen. nov.** is characterized by a shape and structure typical for females (see Fig. 6E in^5^ and Figs. S3a, b, S4a, c).

## Biology

Among the mayfly taxa reported in the Mesozoic, Baetiscidae represent one of the rarest. From all known Baetiscidae fossils, only three specimens represent larvae (two of which are described in this study). Apart from *Koonwarrabaetisca* **gen. nov.**, there is only a single larva of *P. bechlyi* from the Cretaceous Crato Formation^8^.

Larvae of extant Baetiscidae occur in running waters, inhabiting calm sections or stream margins with accumulated detritus, upon which they feed^14^. Fossil Baetiscidae probably exhibited similar biology, as already assumed by Staniczek et al.^5^. These authors considered the relative rareness of *P. bechlyi*, found as a single larva within the Crato Formation, a site otherwise rich in fossil mayflies, as an indication that the species was allochtonous to the depositional site and probably lived upstream. A similar situation exists in Koonwarra, where hundreds of mayfly larvae have been discovered^1^, with only two *Koonwarrabaetisca* **gen. nov.** larvae found. We studied numerous additional material housed in the collection of Museum Victoria and not investigated by Jell and Duncan^1^; vast majority of this material contains only *Australurus plexus* Jell & Duncan, 1986 and no further specimens of *Koonwarrabaetisca* **gen. nov.** were discovered.

Therefore, we assume *Koonwarrabaetisca* **gen. nov.** also represented allochtonous specimens that lived upstream and were only carried to the shallow freshwater lake forming the deposition site. Such taxa living in upstream sections are generally very rarely found^15^, which corresponds with the rareness of both *Koonwarrobaetisca* **gen. nov.** and *P. bechlyi*. Nevertheless, a relatively intact state of *Koonwarrobaetisca* **gen. nov.** larvae, with most legs and caudal filaments preserved, suggests the transport to the deposition site was relatively short. Furthermore, we noted that both larvae of *Koonwarrabaetisca* **gen. nov.** fossilised in lateral position, which indicates their body was not significantly expanded laterally and without large lateral spines, contrary to most Baetiscidae.

Other peculiarities of *Koonwarrabaetisca* **gen. nov.** are pronotum at least partially separated from mesonotum and elongated leg segments, especially femora and tarsi. This could provide higher mobility of the thoracic section, which in combination with a more movable abdominal segments lacking posterolateral and posteromedian projections indicates the larvae were adapted for active and presumably rapid movement in the littoral habitats of streams or rivers rich of water plants and detritus.

### Biogeography

**Figure 4:**
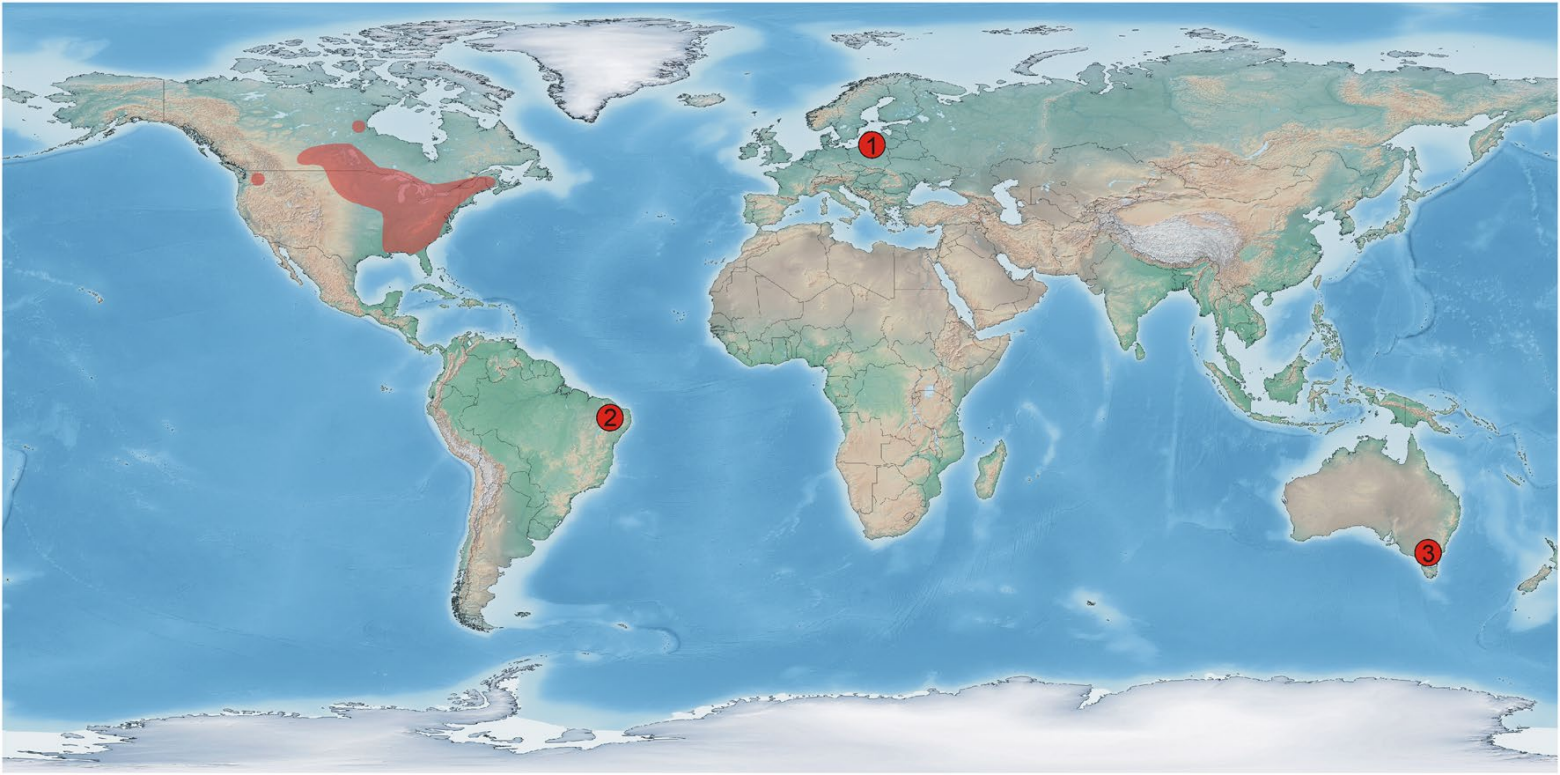
Map showing worldwide distribution of Recent and fossil Baetiscidae. Fossil sites marked as numbered red dots. 1: Baltic amber; 2: Crato Formation; 3: Koonwarra Fossil Bed. Light coloured polygons in North America represent Recent distribution (according to Berner et al.^6^). Map generated using SimpleMappr^39^.

**Figure 5:**
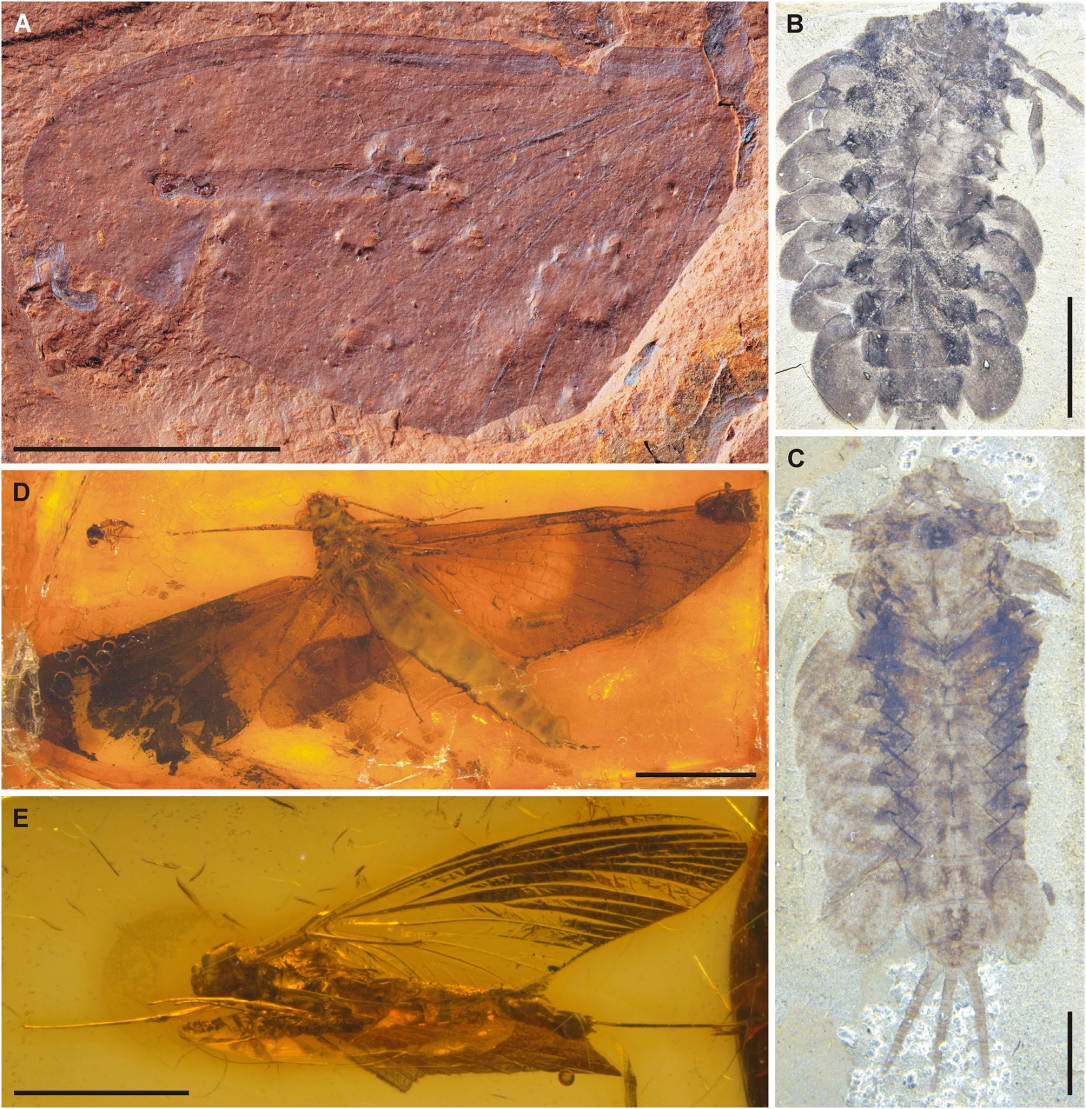
Mayfly taxa with Pangean origin: fossil representatives of Posteritorna *incertae sedis* (**A**) and Ameletopsidae (**B**–**E**). (**A**) isolated forewing of adult, Middle Miocene*,* McGraths Flat Konservat-Lagerstätte, New South Wales, Australian Museum in Sydney (Australia), scale bar = 5 mm; (**B**) *Promirara cephalota* Jell & Duncan, 1986, holotype, larva, NMVP102472 A, Early Cretaceous Koonwarra Fossil Bed, Museum Victoria, Melbourne (Australia), scale bar = 5 mm; (**C**) *Promirara cephalota* Jell & Duncan, 1986, paratype, larva, NMVP102473 A, the same site and collection as holotype, scale bar = 2 mm; (**D**) *Balticophlebia henningi* Demoulin, 1968, holotype, female imago, reg. no. 36, Eocene Baltic amber, Kaliningrad, Russian Federation, Geowissenschaftliches Zentrum, Universität Göttingen (Germany), scale bar = 5 mm; (**E**) Ameletopsidae, gen. et sp. indet., female imago, Do-1268-K, Miocene Dominican amber, Dominican Republic, State Museum of Natural History, Stuttgart (Germany), scale bar = 2 mm.

Attribution of *Koonwarrabaetisca* **gen. nov.** to Baetiscidae confirms almost worldwide distributional range of this family in deep time (Fig. 4). From the Cretaceous, their fossils were found in Australia and South America^8^. Later findings represent Eocene amber inclusions from Europe and Russia^5,16,17^. Given the fact that the distributional range of Baetiscidae throughout their history is documented on four continents, the family most probably existed already prior to the main continental breakout, diversified and dispersed overland to several of today's continents probably during Early Jurassic at latest. This scenario was already hypothesized by Pescador et al.^9^, who assumed Pangean origin and approximate age of Baetiscidae at least 200 My. Later long-distance transcontinental dispersal of this mayfly lineage is unlikely since the larvae live exclusively in freshwater and the winged stages are generally short-lived and poor fliers^14,18^. Similar pattern of ancient lineages which already existed and dispersed across Pangea and today occur only in a small part of their original area was reported in other aquatic insects, such as stoneflies of the family Notonemouridae^19^. Within mayflies, a similar situation can be mentioned for the family Ameletopsidae (Fig. 5), with the current distributional pattern restricted to Australia, New Zealand and Chile-Patagonian Region of South America^7^. Its fossil representatives have been reported from the Eocene Baltic amber, Cretaceous Koonwarra Fossil Bed^1,7^, and finally yet undescribed species putatively attributed to the same family was discovered in the Miocene Dominican amber (Arnold H. Staniczek, *personal communication*).

The oldest known representatives of Baetiscidae are dated in late Early Cretaceous^5^. However, several related lineages of Posteritorna (Mesozoic family Sharephemeridae Sinitshenkova, 2002) are reported already from different parts of Pangea during the Triassic and Early Jurassic^10,12,20,21^. It is possible that the Jurassic Baetiscidae have not yet been discovered given their general rarity in the fossil record. The data on the historical biogeography of the family Baetiscidae correlate well with the results of the cladistic analysis obtained here (Fig. 3). All extant and fossil representatives of Baetiscinae are restricted to the Northern Hemisphere, whereas Protobaetiscinae **subfam. nov.** occurred exclusively in Gondwana. Therefore, we assume that the separation of Baetiscinae and Protobaetiscinae **subfam. nov.** resulted from a vicariant event connected with the break-up of Pangea into Laurasia and Gondwana during the Jurassic.

Considering the current distributional range restricted to the Nearctic realm, Baetiscidae must have been subject of several local extinctions outside Nearctic. Staniczek et al.^5^ estimated timing of some of these extinctions. The extinction of *Protobaetisca* in South America was assumed to take place during the K/Pg event. Nevertheless, it could have occurred even earlier, since a period of highly increased extinction rate in mayflies was found at about mid-Cretaceous, although this extinction was more catastrophic for lacustrine taxa^22^. In any case, it seemed to have much higher impact on aquatic insects than the later K/Pg event^23–25^. The time of Baetiscidae extinction in Australia is unknown. There are no mayfly taxa attributable to Baetiscidae in the current Australian fauna, and only a single extant species of Prosopistomatidae (*Prosopistoma pearsonorum* Campbell & Hubbard, 1998) is known from Northern Australia^7^. Additionally, among a few mayfly fossils discovered as a part of an exceptionally well-preserved rainforest Miocene biota of the New South Wales, almost complete isolated forewing of a mayfly has been found, attributable to Posteritorna (Arnold H. Staniczek personal communication; for more details on the locality see McCurry et al.^26^). Judging from the size and shape of the wing plate and characters of venation, this fossil is not congeneric with *Koonwarrabaetisca* **gen. nov.** and belongs to a new undescribed genus and species (Fig. 5). Therefore, a few Posteritorna lineages evidently still occurred in Australia during Miocene.

As for the extinction of *Balticobaetisca*, recorded in the Palaearctic during Eocene, Pescador et al.^9^ hypothesized the role of Pleistocene glaciations, based on previous considerations of Lehmkuhl^27^ and Pescador and Berner^13^. The same fate evidently also befell the fossil representatives of the genera *Analetris* Edmunds, 1972 and *Siphloplecton* Clemens, 1915, which are described from the Eocene amber of Europe and known as extant species only from North America^9,28–30^.

## Material and methods

### Locality and geological setting

Discovered in 1961 the Early Cretaceous Koonwarra Fossil Bed is representing a short succession of varved lake deposits within a thick fluviatile succession of the Korumburra Group. Together with most abundant fish, lacustrine sequences contain the bed with a rich and diverse insect fauna that lived in the lake and the streams flowing into it^31^.

Thus, all mayfly material studied by Jell and Duncan^1^ and referred here, originates from this one locality, although from several different levels within the 5 m bed. At first it was assumed that the sediments belong to late Aptian^32^. Primary description of the geological setting, the age, and the palaeoecology were provided by Waldman^33^, with detailed data on fish fauna under the name "Koonwarra fish-bed"^1^. Later, Dettmann^34^ and Seegets-Villiers and Wagstaff^35^ interpreted the Koonwarra Fossil Bed as Barremian–Aptian based on *Cyclosporites hughesii* Zone palynomorphs. The age of the Bed was dated to 118 ± 5–115 ± 6 Ma based on fission track dating of Koonwarra samples, and palynologically corresponds to the middle–late Aptian^4,31,34^.

### Material

All specimens originating from the Early Cretaceous Koonwarra Fossil Bed are deposited in the Museum Victoria, Melbourne, Australia under the accession numbers NMVP103209 (part [A] and counterpart [B] of the holotype of *Koonwarrabaetisca duncani* **sp. nov.**) and NMVP103210 (part [A] and counterpart [B] of the holotype of *Koonwarrabaetisca jelli* **sp. nov.**). These specimens represent compression fossils.

The holotype of *Protobaetisca bechlyi* Staniczek, 2007 (unknown outcrops in the vicinity of Nova Olinda municipality, Ceará State, Brazil), is housed in the fossil collection of the State Museum of Natural History Stuttgart, Germany [SMNS] under the accession number SMNS 66620 (see also Staniczek^8^). Putative adult specimen of this species as designated by Staniczek et al.^5^ is housed in Senckenberg Naturmuseum Frankfurt, Germany [SMF], under inventory number SMF VI 993. Both specimens of the genus *Protobaetisca* Staniczek 2007 undoubtedly belong to the Nova Olinda Member of the Crato Formation (upper Aptian, Early Cretaceous).

Comparative larval material of the extant species of the genus *Baetisca* Walsh, 1863 from USA is housed in the Institute of Entomology, Biology Centre CAS [IE] and SMNS was comprised of the following taxa: *Baetisca bajkovi* Neave, 1934, *B. becki* Schneider & Berner, 1963, *B. berneri* Tarter & Kirchner, 1978, *B. lacustris* McDunnough, 1932, *B. laurentina* McDunnough, 1932, *B. rogersi* Berner, 1940 and *B. rubescens* Provoncher, 1878.

### Optical equipment, measurements, terminology

The material was examined dry and under a film of ethyl alcohol using stereomicroscopes Olympus SZX7 and Leica M205C. The photographs were taken using a Canon EOS 550D digital camera equipped with MP-E 65 mm and EF-S 60 mm macro-lenses. Original photographs were processed using image-editing software and some were processed by the stacking software Helicon Focus Pro (Helicon Soft, Kharkiv, Ukraine) or Zerene Stacker (Zerene systems LLC, Richland, USA). Photographs were sharpened and the contrast and tonality adjusted using Adobe Photoshop™ version CS6 (Adobe Systems Incorporated, San Jose, USA).

Serial photographs of the extant species of *Baetisca* and holotype of *P. bechlyi* with different focal planes were taken in SMNS through a Leica Z16 APO Macroscope equipped with a Leica DFC450 Digital Camera using Leica Application Suite v. 3.1.8. Resulting photo stacks were processed with Helicon Focus Pro 6.4.1 to obtain combined photographs with extended depth of field.

For scanning electron microscopy (SEM) the larvae of extant species *B. rogersi* were used. All parts were subsequently dehydrated through a stepwise immersion in ethanol, dried by critical point drying (Leica EM CPD300), and mounted on SEM stubs. The mounted material was coated with a 5 nm Au/Pd layer (Leica EM ACE200) and subsequently examined and photographed with a Zeiss EVO LS 15 scanning electron microscope. All photographs were subsequently sharpened and adjusted in contrast and tonality in Adobe Photoshop™ CC2019. Some scanning electron micrographs were taken using Apreo SEM (Thermo Fisher Scientific) in the Biology Centre CAS. The unprocessed sample was observed under Hi-vacuum conditions. Imaging parameters were as follows: accelerating voltage—5 kV, current—50 pA, working distance—16 mm. ETD detector was used to collect the signal.

The measurements of individual body parts were inferred from the photographs taken with a calibration scale or ocular grid (Table 1). Anatomical larval terminology, characters and nomenclature of wing veins used throughout the text follow^7,9,13,36,37^.

### Nomenclatural acts

The electronic edition of this article conforms to the requirements of the amended International Code of Zoological Nomenclature, and hence the new names contained herein are available under that Code from the electronic edition of this article. This published work and the nomenclatural acts it contains have been registered in ZooBank, the online registration system for the ICZN. The ZooBank LSID for this publication is: urn:lsid:zoobank.org:pub:2C4203AC-441C-4148-82DB-24F5C588D805.

### Phylogenetic analysis

The matrix of morphological characters was compiled based on Staniczek et al.^5^ and Pescador et al.^9^, with two newly introduced characters. For the matrix and the list of characters, see Supplementary Tables S1 and S2 in Supplementary Information 2. The dataset contained 38 characters (numbered from 0 to 37) and has been coded for 16 taxa. Both species of *Koonwarrabaetisca* **gen. nov.** were treated as a single terminal, since they did not differ in any character used in the matrix.

The maximum parsimony analysis was conducted using TNT^38^. The analysis settings were the same as used by Staniczek et al.^5^. All characters were treated as non-additive and unordered. An exhaustive search was run under implicit enumeration command (collapsing rule applied) and implied weights, testing several concavity constant values (k = 1–20). Traditional and New Technology searches were also conducted for comparison. To estimate support of nodes, the Relative Bremer Support was calculated. Trees suboptimal by 15 steps were retained, resulting in 1357 trees. The bootstrap support was also calculated with 1000 pseudoreplicates, using the implicit enumeration search.

## Conclusion

A new extinct mayfly genus *Koonwarrabaetisca* **gen. nov.**, is described based on re-examination of larval imprints from the Early Cretaceous Koonwarra Fossil Bed in Australia. These larvae have been occasionally referred to in the contributions describing the fossil fauna of Australia, as well as in investigations of extinct and extant Baetiscidae and Posteritorna in general. However, no strong evidence for their systematic affiliation to Baetiscidae has been previously presented. We provide a detailed reconstruction and description of larval characters, indicating that the new genus belongs to the family Baetiscidae. These include the shape and venation of forewing remnants with presumably posteritornous condition and nearly rounded hind wing, robust mesonotal shield covering abdominal terga I–VI and forming a gill chamber, and specific shape and arrangement of tracheal gills inside of the shield. This new genus comprises two new species *Koonwarrabaetisca jelli* **sp. nov.** and *K. duncani* **sp. nov.**, which are markedly differing by the presence of prominent posterolateral projection of abdominal segment IX in the latter species. We also conclude that the Cretaceous Baetiscidae represented by genera *Koonwarrabaetisca* **gen. nov.** and *Protobaetisca* should be assigned to the separate taxon, namely the subfamily Protobaetiscinae **subfam. nov.** established here, primarily based on markedly shortened thoracic sterna (observed in both larvae and putative adult), and absence of prominent posterolateral projections and median spines on abdominal segments VI–VIII of larvae.

Based on the information on taphonomy of fossil materials from Koonwarra Fossil Bed and morphological structure of *Koonwarrabaetisca* **gen. nov.** larvae, we concluded that they represented an allochtonous element inhabiting the upper sections of running waters. The structure of the thorax, legs and abdominal segments indicate that the larvae were adapted to more active swimming in the littoral habitats of streams or rivers rich in water plants and detritus.

The description of *Koonwarrabaetisca* **gen. nov.** in Baetiscidae finally clarifies the question of Pangean origin of this family. Moreover, the whole set of data on historical biogeography of the family is well correlated with the results of the cladistic analysis. It can be assumed that the separation of Baetiscinae and Protobaetiscinae **subfam. nov.** is linked to the break-up of Pangea into Laurasia and Gondwana in the Jurassic.

### Supplementary Information

Supplementary Information.

Supplementary Information.

## Data Availability

All data generated or analysed during this study are included in this published article and its Supplementary Information files. All relevant data are available from the authors. The datasets generated during and/or analysed during the current study are available from the corresponding author upon reasonable request. Two fossils used in this study are housed as part and counterpart specimens in the public collection of the Museum Victoria (Melbourne, Australia); other information about origin of the material is specified in the Section “Material and methods”. Respective inventory numbers of studied specimens are listed in this published article. Detailed information about fossil invertebrate collection from the Early Cretaceous Koonwarra Fossil Bed is available under web-link: https://collections.museumsvictoria.com.au/search?locality=Koonwarra (September 1, 2023). Requests for access to the fossil materials should be addressed to the curator of the collection. This work has been registered online at zoobank.org under LSID urn:lsid:zoobank.org:pub:2C4203AC-441C-4148-82DB-24F5C588D805. The new taxa are registered in Zoobank.org.
