# Supplementary material for: A new mayfly subfamily sheds light on the early evolution and Pangean origin of Baetiscidae (Insecta: Ephemeroptera)
Source: Sci Rep. 2024 Jan 18;14:1599. doi: 10.1038/s41598-024-51176-7 (PMC10796926; doi:10.1038/s41598-024-51176-7)
Supplement: Supplementary file 1 — Supplementary Information. [file 41598_2024_51176_MOESM1_ESM.pdf]

Supplementary Information 1
Figure S1

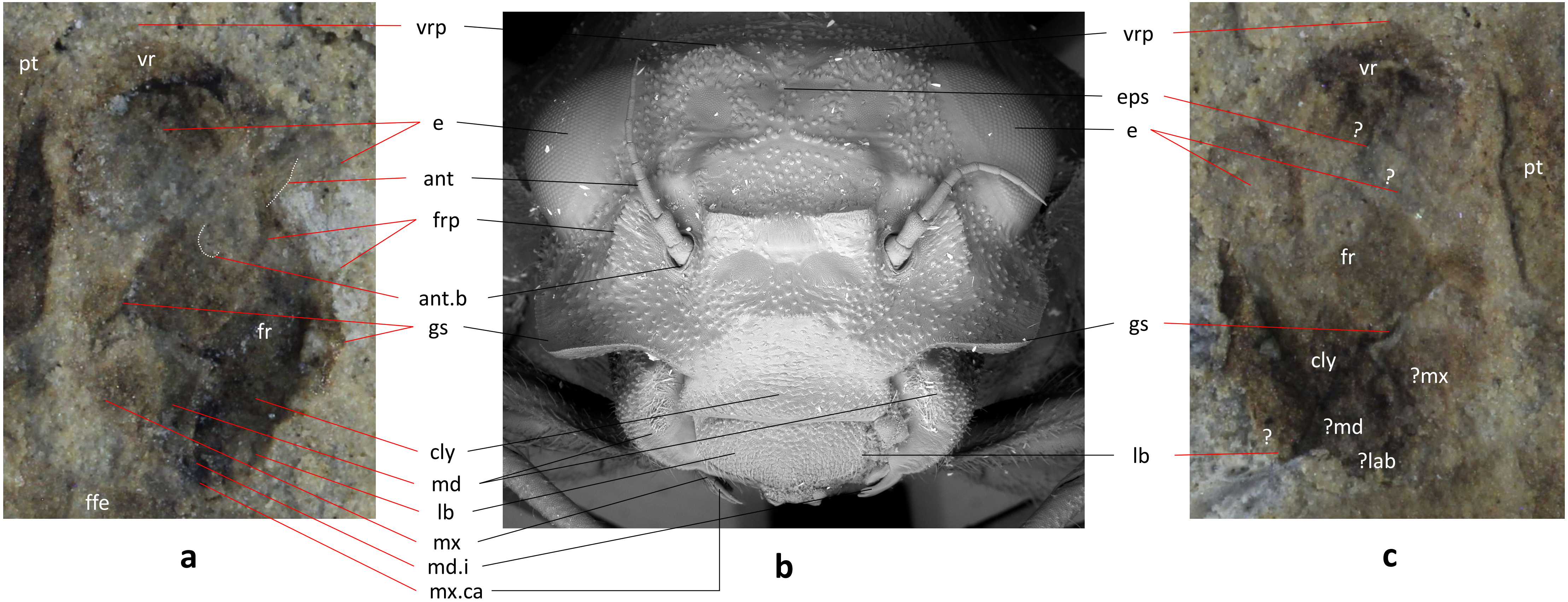

Supplementary Information 1
Figure S2

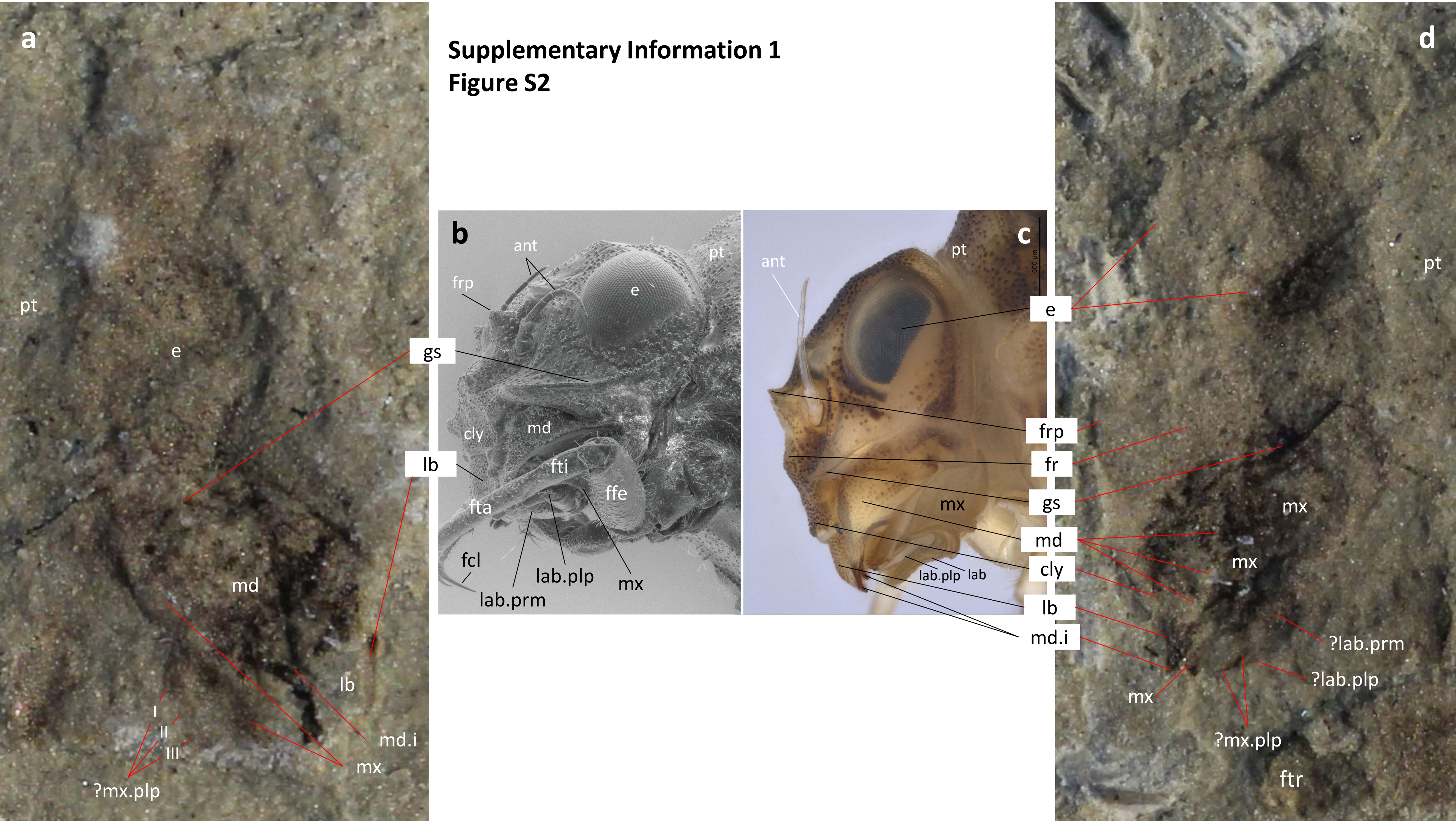

Supplementary Information 1

Descriptions of Figures S1–S2

Figure S1. *Koonwarrabaetisca jelli* **gen. et sp. nov.**, holotype (**a**, **c**) and comparative Recent material of *Baetisca rogersi* Berner, 1940, USA, coll. SMNS (**b**).
(**a**) Specimen P103210A, general view of head with interpretation of individual body parts; (**b**) *Baetisca rogersi*, general view of head with interpretation of individual body parts under ESEM; (**c**) Specimen P103210B, general view of head with interpretation of individual body parts.
Interpretation of larval antenna on Figure S1a is indicated by white dotted lines.
Without scales.

Figure S2. *Koonwarrabaetisca duncani* **gen. et sp. nov.**, holotype (**a**, **d**) and comparative Recent material of *Baetisca rogersi* Berner, 1940, USA, coll. SMNS (**b**, **c**).
(**a**) Specimen P103209B, general view of head with interpretation of individual body parts; (**b, c**) *Baetisca rogersi*, general view of head with interpretation of individual body parts under ESEM [**b**] and macrophotography [**c**]; (**d**) Specimen P103209A, general view of head with interpretation of individual body parts.
Without scales.

Abbreviations for Figures S1 and S2
Head: ant – antenna; ant.b – base of antenna; cly – clypeus; e – eye; fr – frons; frp – frontal protuberance; gs – genal shelf; lab – labium; lab.plp – labial palp; lab.prm – prementum (labium); lb – labrum; md – mandible; md.i – mandibular incisors; mx – maxilla; mx.ca – maxillary canines; mx.plp – maxillary palp (I, II, III – respective segments of maxillary palp); vr – vertex; vrp – vertex protuberance.
Thorax: pt – prothorax; ftr – foretrochanter; ffe – forefemur; fti – foretibia; fta – foretarsus; fcl – foreclaw.

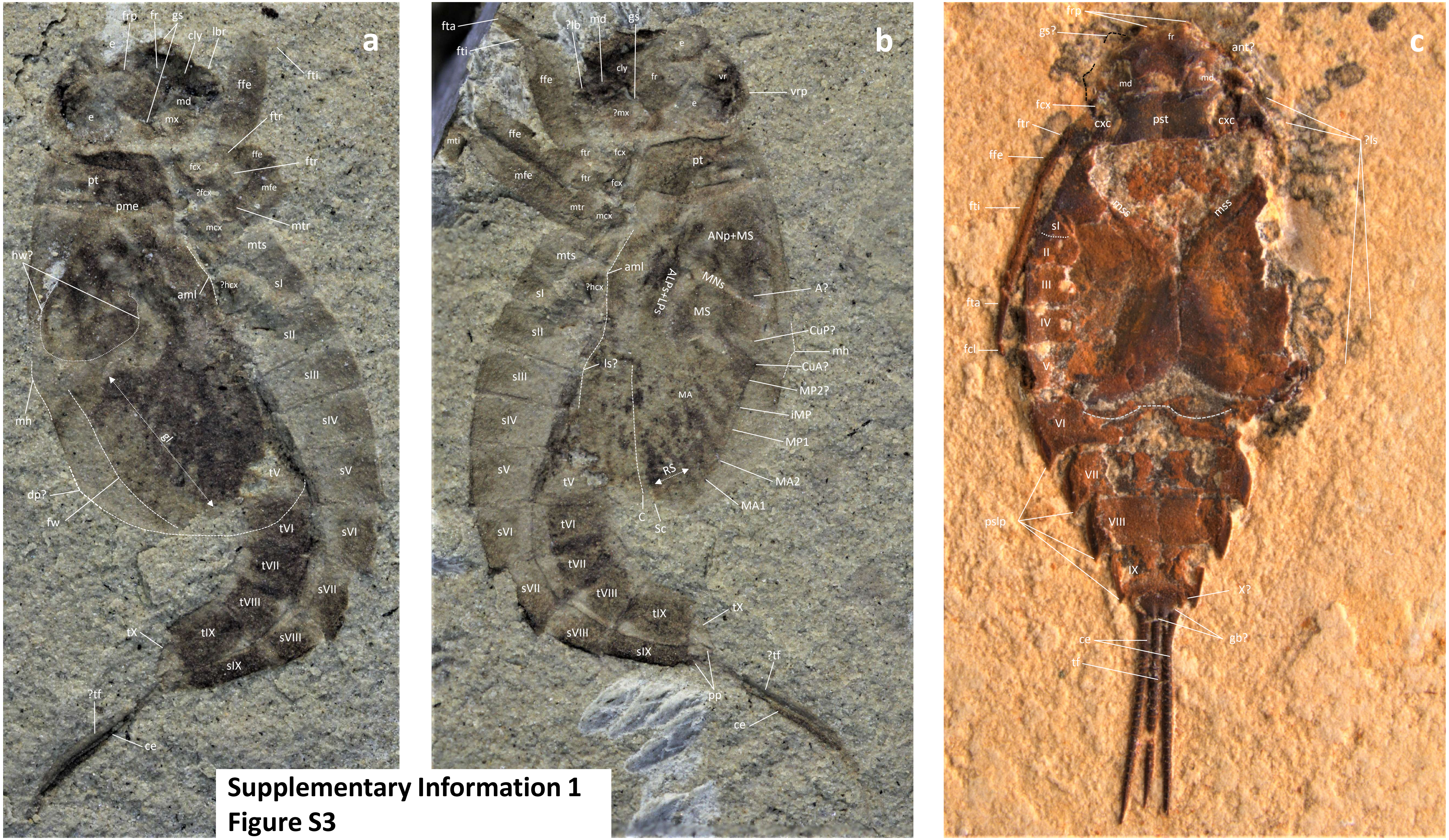

Supplementary Information 1
Figure S3

Supplementary Information 1

Descriptions of Figures S3

Figure S3. *Koonwarrabaetisca jelli* **gen. et sp. nov.**, holotype (**a**, **b**) and *Protobaetisca bechlyi* Staniczek, 2007, holotype, Lower Cretaceous, Upper Aptian, Crato Formation, coll. SMNS 66620 (**c**).
(**a**) Specimen P103210A, general lateral view with interpretation of individual body parts; (**b**) Specimen P103210B, general lateral view with interpretation of individual body parts; (**c**) *Protobaetisca bechlyi* Staniczek, 2007, holotype, general ventral view with interpretation of individual body parts.
Interpretation of larval wing pad shape and venation, and mesonotal shield shape and structure are indicated by white dotted lines.
Without scales.

Abbreviations for Figure S3
Head: cly – clypeus; e – eye; fr – frons; frp – frontal protuberance; gs – genal shelf; lab – labium; lb – labrum; md – mandible; mx – maxilla; vr – vertex; vrp – vertex protuberance.
Thorax: amh – anteromedian hump; aml – anteromedian lobe; cxc – coxal cavity; dp – dorsal projection; fw – forewing; hw – hind wing pad lml – lateromedian lobe; ls – lateral spine; mdd – middorsal depression; mh – medial hump; mss – medioscutal suture; mst – mesothorax; mts – metasternum; pme – protonal-mesonotal elevation; psme – posteromedian elevation; pst – prosternum; pt – prothorax; ALPs+LPs – anterolateroparapsidal suture and lateroparapsidal suture; ANp+MS - anteronotal protuberance and medioscutum; MNs – mesonotal suture;
C – costa; Sc – subcosta; RS – radius sector; MA (MA1, MA2) – media anterior sector; MP (MP1, iMP, MP2) – media posterior sector; CuA – cubitus anterior; CuP – cubitus posterior; A – anal vein.
fcx – forecoxa; ftr – foretrochanter; ffe – forefemur; fti – foretibia; fta – foretarsus; mcx – middle coxa; mtr – middle trochanter; mfr – middlefemur; mti – middle tibia; hcx – hind coxa
Abdomen: gl – gills; pslp – posterolateral projection; tI–X – terga I–X; sI–IX – sterna I–IX; gb – genital bud; pp – paraproct plates; tf – terminal filament [paracercus] ce – cerci.

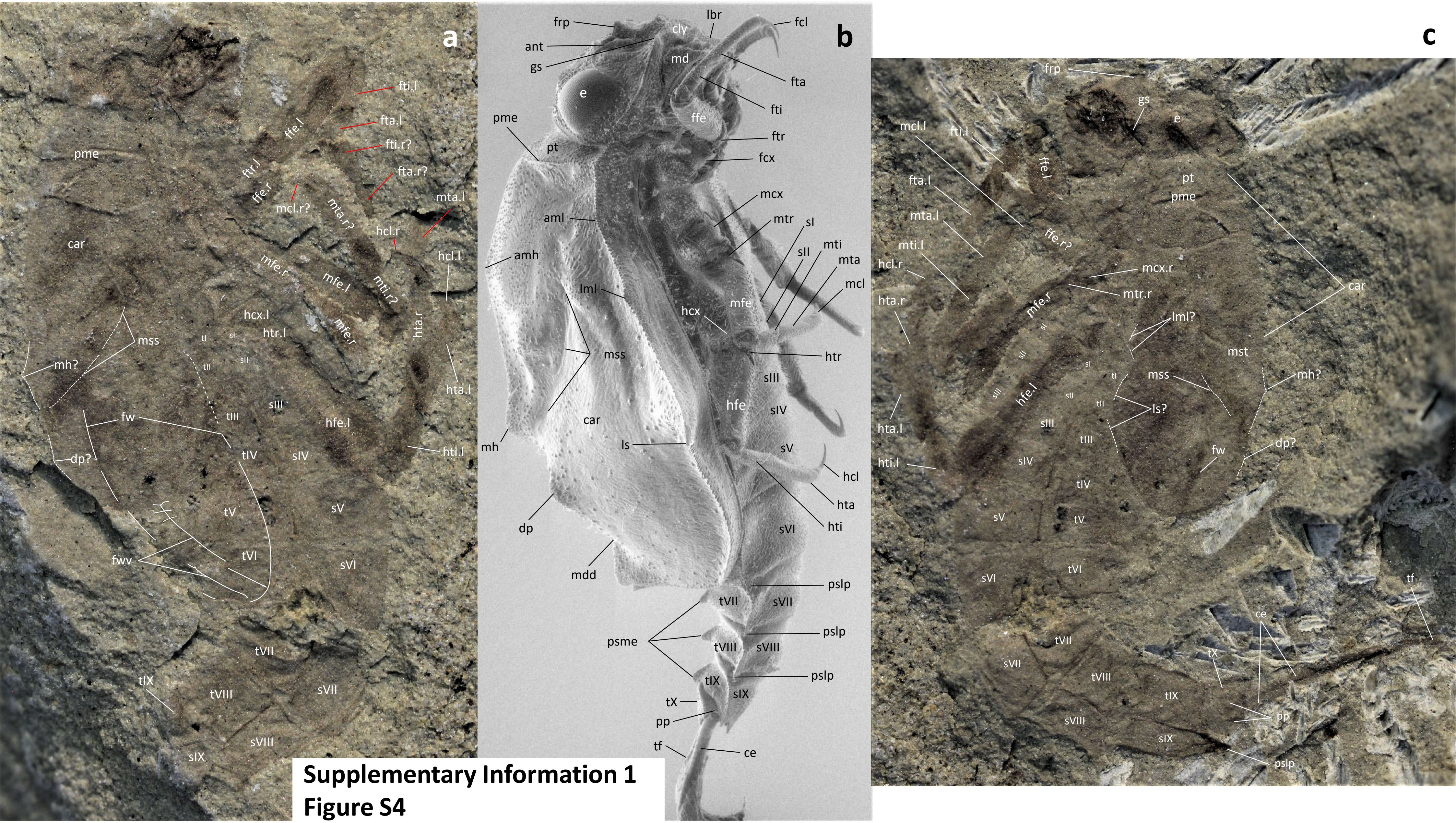

Supplementary Information 1
Figure S4

Supplementary Information 1

Descriptions of Figures S4

Figure S4. *Koonwarrabaetisca duncani* **gen. et sp. nov.**, holotype (**a**, **c**) and comparative Recent material of *Baetisca rogersi* Berner, 1940, USA, coll. SMNS (**b**).
(**a**) Specimen P103209B, general lateral view with interpretation of individual body parts; (**b**) *Baetisca rogersi*, general lateral view with interpretation of individual body parts; (**c**) Specimen P103209A, general lateral view with interpretation of individual body parts.
Interpretation of larval wing pad shape and venation, and mesonotal shield shape and structure are indicated by white dotted lines.
Without scales.

Abbreviations for Figure S4
Head: ant – antenna; cly – clypeus; e – eye; frp – frontal protuberance; gs – genal shelf; lb – labrum; md – mandible.
Thorax: amh – anteromedian hump; aml – anteromedian lobe; dp – dorsal projection; fw – forewing; lml – lateromedian lobe; ls – lateral spine; mdd – middorsal depression; mh – medial hump; mss – medioscutal suture; mst – mesothorax; pme – protonal-mesonotal elevation; psme – posteromedian elevation; pt – prothorax; fwv – remnants of forewing venation;
fcx – forecoxa; ftr – foretrochanter; ftr.l – foretrochanter [left]; ffe.l – forefemur [left]; ffe.r – forefemur [right]; fti.l – foretibia [left]; fti.r – foretibia [right]; fta.l – foretarsus [left]; fta.r – foretarsus [right]; mcx – middle coxa; mcx.r – middle coxa [right]; mtr – middle trochanter; mtr.r – middle trochanter [right]; mfe.l – middle femur [left]; mfe.r – middle femur [right]; mti.l – middle tibia [left]; mti.r – middle tibia [right]; mta.l – middle tarsus [left]; mta.r – middle tarsus [right]; mcl.r – middle claw [right]; hcx – hind coxa; hcx.l – hind coxa [left]; htr – hind trochanter; htr.l – hind trochanter [left]; hfe – hind femur; hfe.l – hind femur [left]; hti – hind tibia; hti.l – hind tibia [left]; hti.r – hind tibia [right]; hta – hind tarsus; hta.l – hind tarsus [left]; hta.r – hind tarsus [right]; hcl – hind claw; hcl.l – hind claw [left]; hcl.r – hind claw [right].
Abdomen: pslp – posterolateral projection; tI–X – terga I–X; sI–IX – sterna I–IX; pp – paraproct; tf – terminal filament [paracercus]; ce – cerci.
