# Supplementary material for: A new mayfly subfamily sheds light on the early evolution and Pangean origin of Baetiscidae (Insecta: Ephemeroptera)
Source: Sci Rep. 2024 Jan 18;14:1599. doi: 10.1038/s41598-024-51176-7 (PMC10796926; doi:10.1038/s41598-024-51176-7)
Supplement: Supplementary file 2 — Supplementary Information. [file 41598_2024_51176_MOESM2_ESM.pdf]

Supplementary Information 2

Supplementary Table S1. Morphological characters and their states coded for phylogenetic analysis (see Supplementary Table 2); evolved from [5] and [9], except where noticed.

Character	Character states
Nymph	
0. Frontal projection	(0) absent (1) bilobed (2) non-bilobed
1. Genae	(0) not produced into spines (1) produced into spines
2. Genal shelf	(0) not projected/less projected (1) projected
3. Antennae	(0) short (1) long
4. Compound eyes	(0) non-stripped (1) stripped
5. Occipital tubercles (female)	(0) absent (1) present
6. Mesonotal shield	(0) smooth (1) with projections and spines
7. Lateral margins of mesonotal shield	(0) strongly divergent (1) nearly parallel
8. Lateromedian lobe of mesonotal shield	(0) absent (1) weakly developed broadly rounded (2) well-developed subtriangular
9. Lateral projections of mesonotal shield	(0) absent (1) length subequal to shorter than width at base (2) length 1.3-1.7x width at base (3) length 2x or more the width at base
10. Dorsal projections of mesonotal shield	(0) absent (1) present, low elevated, broadly rounded (2) present, highly elevated, cone or peg shaped
11. Lateral margins of mesonotal shield	(0) smooth (1) finely denticulate (2) coarsely serrate
12. Outlining/markings of ventral margin of mesonotal shield	(0) absent (1) present
13. Length vs width of mesonotal shield	(0) length subequal to or shorter than width (1) length longer than width
14. Pattern of speckling/ markings on mesonotal shield	(0) banded (1) reticulate (2) finely checked or grainy (3) dotted (4) smooth/without pattern
15. Apex of lateral projections of mesonotal shield	(0) absent (1) blunt (2) tapered to pointed, if sclerotised, only at tip (3) pointed and heavily sclerotised
16. Legs	(0) unicoloured (1) bicolored
17. Tarsal claws	(0) <1/2 length of tarsi (1) >1/2 length of tarsi
18. Lateral margins of abdomen	(0) glabrous or with few short hairs (1) with long thick hairs
19. Posterolateral projections of abdominal segments VI-VIII	(0) weakly developed, apically blunt (1) well-developed, apically sharp pointed, (3) absent
20. Dorsoventral shape of nymph	(0) compressed (1) distinctly convex
21. Pronotum and mesonotal shield partially separated by suture (new)	(0) present (1) absent
22. Length of thoracic sterna (new)	(0) Less than 0.25x body length; (1) more than 0.25x body length
Adult	
23. Shape of penis lobes	(0) triangular (1) subquadrangular
24. Penis lobes	(0) separated (1) not separated
25. First segment of forceps	(0) with triangular projection on inner margin (1) without triangular projection on inner margin
26. Ratio forewing/hind wing	(0) almost 1/2 (1) 1/3 (2) 1/4 (3) 1/5
27. Forewing colouration of imago	(0) entirely hyaline (1) basal 1/3-1/2 flushed with orange to reddish brown
28. Forewing shape	(0) triangular (1) elongated/narrower
29. Cross veins of forewing (new)	(0) absent (1) present - prominent (2) present - not prominent
30. MA vein of forewing (new)	(0) unforked (1) forked
31. Hind wing colouration of imago	(0) entirely hyaline (1) basal 1/4-3/4 flushed with orange to reddish brown (2) almost entirely orange to reddish brown
32. Shape of hind wing	(0) oval (1) rounded
33. Costal projection on hind wing	(0) absent (1) present
34. Middorsal transverse elevation in tergum VI	(0) absent (1) present
35. Apical cleft in sternum IX (female)	(0) absent (1) present
36. Prosternal bispinate projection	(0) absent (1) present
37. Each foreleg in male adult with	(0) two blunt claws (1) single blunt claw

Supplementary Table S2. Matrix of morphological characters and states used for the phylogenetic analyses.

Taxa of Posteritorna	Characters																																					
	**0**	**1**	**2**	**3**	**4**	**5**	**6**	**7**	**8**	**9**	**10**	**11**	**12**	**13**	**14**	**15**	**16**	**17**	**18**	**19**	**20**	**21**	**22**	**23**	**24**	**25**	**26**	**27**	**28**	**29**	**30**	**31**	**32**	**33**	**34**	**35**	**36**	**37**
Prosopistoma variegatum	0	0	0	0	0	0	1	0	0	0	0	0	0	0	0	0	0	0	0	0	0	1	1	0	0	1	0	0	0	0	0	0	0	1	0	0	0	1
Cretomitarcys luzzii	?	?	?	?	?	?	?	?	?	?	?	?	?	?	?	?	?	?	?	?	?	?	?	?	?	?	0	?	0	1	1	?	0	0	?	?	?	0
Koonwarrabaetisca spp.	2	0	0	0	0	0	1	1	1	1	?	0	?	?	4	1	1	0	?	3	1	0	0	?	?	?	?	?	?	?	?	?	?	?	?	?	?	?
Protobaetisca bechlyi	1	0	0	1	?	?	2	0	0	?	?	0	?	?	0	?	1	?	0	?	?	?	0	?	?	?	3	?	0	1	1	?	1	1	?	?	?	?
Balticobaetisca bispinata	?	?	?	?	?	?	?	?	?	?	?	?	?	?	?	?	?	?	?	?	?	?	?	0	0	0	1	1	1	1	1	?	1	1	0	?	1	0
Balticobaetisca stuttgardia	?	?	?	?	?	?	?	?	?	?	?	?	?	?	?	?	?	?	?	?	?	?	?	0	0	0	1	0	1	?	1	0	1	1	0	?	1	0
Baetisca becki	1	0	1	0	0	1	2	0	1	3	2	0	0	1	0	0	0	1	1	3	3	1	1	0	1	1	1	1	1	?	1	1	1	1	1	1	1	0
Baetisca carolina	2	1	1	0	0	0	2	0	1	2	1	0	0	1	0	0	0	1	0	1	3	1	1	0	1	1	1	1	1	?	1	1	1	1	1	1	1	0
Baetisca columbiana	1	0	1	0	0	1	2	0	0	1	0	0	0	1	0	0	0	1	0	1	1	1	1	?	?	?	?	?	?	?	?	?	?	?	?	?	?	?
Baetisca escambiensis	2	1	1	0	1	0	2	0	1	3	0	0	0	0	1	1	1	0	1	1	3	1	1	1	1	1	?	1	1	?	1	1	1	1	1	1	1	0
Baetisca gibbera	1	0	1	0	0	0	2	0	0	1	0	0	0	1	0	0	0	1	0	1	1	1	1	0	1	1	?	0	1	?	1	0	1	1	1	1	1	0
Baetisca lacustris	1	0	1	0	0	1	2	0	1	2	2	0	0	1	0	0	0	1	1	2	2	1	1	0	1	1	?	0	1	?	1	0	1	1	1	1	1	0
Baetisca laurentina	1	0	1	0	0	1	2	1	1	2	2	0	1	0	0	0	0	1	1	1	2	1	1	0	1	1	2	0	1	?	1	0	1	1	1	1	1	0
Baetisca obesa	1	0	1	0	0	1	2	1	1	2	2	0	0	1	0	0	0	1	1	1	2	1	1	0	1	1	1	0	1	?	1	0	1	1	1	1	1	0
Baetisca rogersi	1	0	1	0	0	1	2	0	2	2	2	1	0	1	0	0	0	1	0	3	3	1	1	0	1	1	1	1	1	2	1	1	1	1	1	1	1	0
Baetisca rubescens	2	1	1	0	0	0	2	0	0	1	0	0	0	1	0	0	0	1	1	1	2	1	1	0	1	1	?	1	1	?	1	2	1	1	1	1	1	0
